# Protocol for the combined immunosuppression & radiotherapy in thyroid eye disease (CIRTED) trial: A multi-centre, double-masked, factorial randomised controlled trial

**DOI:** 10.1186/1745-6215-9-6

**Published:** 2008-01-31

**Authors:** Rathie Rajendram, Richard WJ Lee, Mike J Potts, Geoff E Rose, Rajni Jain, Jane M Olver, Fion Bremner, Steven Hurel, Anne Cook, Rao Gattamaneni, Marjorie Tomlinson, Nicholas Plowman, Catey Bunce, Sandra P Hollinghurst, Laura Kingston, Sue Jackson, Andrew D Dick, Nichola Rumsey, Olivia C Morris, Colin M Dayan, Jimmy M Uddin

**Affiliations:** 1 Moorfields Eye Hospital, City Road, London EC1V 2PD, UK; 2 Department of Clinical Science at South Bristol, University of Bristol, UK; 3 Bristol Eye Hospital, Lower Maudlin Street, Bristol, BS1 2LX, UK; 4 Western Eye Hospital, Marylebone Road, London, NW1 5YE, UK; 5 Department of Neuroophthalmology, Internal Box 142, The National Hospital for Neurology and Neurosurgery, Queen Square, London, WC1N3BG, UK; 6 University College London Hospital, Third Floor East, 250 Euston Road, London, NW1 2PQ, UK; 7 Manchester Royal Eye Hospital, Oxford Road, Manchester. M13 9WH, UK; 8 Christie Hospital, Wilmslow Road, Manchester, M20 4BX, UK; 9 Bristol Haematology and Oncology Centre, Horfield Road, Bristol, BS2 8ED, UK; 10 St Bartholomew's Hospital, West Smithfield, London, EC1A 7BE, UK; 11 Academic Unit of Primary Health Care, Department of Community Based Medicine, University of Bristol, 25 Belgrave Road, Bristol, BS8 2AA, UK; 12 Centre for Appearance Research, University of the West of England, Fishponds, Bristol, BS16 2JP, UK; 13 Henry Wellcome Laboratories for Integrative Neuroscience and Endocrinology, University of Bristol, Dorothy Hodgkin Building, Whitson Street, Bristol, BS1 3NY, UK

## Abstract

**Background:**

Medical management of thyroid eye disease remains controversial due to a paucity of high quality evidence on long-term treatment outcomes. Glucocorticoids are known to be effective initially but have significant side-effects with long-term use and recrudescence can occur on cessation. Current evidence is conflicting on the efficacy of radiotherapy and non-steroid systemic immunosuppression, and the majority of previous studies have been retrospective, uncontrolled, small or poorly designed.

The Combined Immunosuppression and Radiotherapy in Thyroid Eye Disease (CIRTED) trial was designed to investigate the efficacy of radiotherapy and azathioprine in *combination *with a standard course of oral prednisolone in patients with *active *thyroid eye disease.

**Methods/design:**

Patients with active thyroid eye disease will be randomised to receive (i) azathioprine *or *oral placebo and (ii) radiotherapy *or *sham-radiotherapy in this multi-centre, factorial randomised control trial. The primary outcome is improvement in disease severity (assessed using a composite binary measure) at 12 months and secondary end-points include quality of life scores and health economic measures.

**Discussion:**

The CIRTED trial is the first study to evaluate the role of radiotherapy and azathioprine as part of a long-term, combination immunosuppressive treatment regime for Thyroid Eye Disease. It will provide evidence for the role of radiotherapy and prolonged immunosuppression in the management of this condition, as well as pilot data on their use in combination. We have paid particular attention in the trial design to establishing (a) robust placebo controls and masking protocols which are effective and safe for both radiotherapy and the systemic administration of an antiproliferative drug; (b) constructing effective inclusion and exclusion criteria to select for active disease; and (c) selecting pragmatic outcome measures.

**Trial registration:**

Current controlled trials ISRCTN22471573

## Background

Thyroid eye disease (TED) can be a visually disabling and cosmetically disfiguring condition which significantly impairs health-related quality of life [1-3]. A wide variety of immunosuppressive therapies have been used to target the early active inflammatory phase of the disease, including glucocorticoids (GC's), radiotherapy, antiproliferative agents, T-cell inhibitors and, more recently, biologics. The goal of these interventions is to suppress the autoimmune inflammatory phase of the disease, thereby altering the course of the disease and reducing the severity of residual changes in the extraocular muscles, orbital fat and other periocular soft tissues which result in permanent visual and cosmetic dysfunction [1,4,5].

GC's have been used to treat TED for over forty years, and several studies have demonstrated their efficacy with an overall response rate of 63–77% [1]. However, GC treatment is typically discontinued after 3 to 5 months [6-9] because of the side-effects associated with their long-term use, and subsequent disease recrudescence is a common problem [10]. This recurrence may be prevented by the concomitant use of ciclosporin [11] (which continues after GC treatment stops), and combinations of ciclosporin and prednisolone also achieve a better initial treatment response than either agent alone [6]. However, the routine use of such second-line immunosuppressive drugs in the management of TED has been limited by fears about their potential toxicity, and glucocorticoid monotherapy remains the mainstay of conventional treatment.

Radiotherapy is also well established in the treatment of TED [12,13], and its efficacy was affirmed in 1993 by the results of a prospective double-blind randomised control trial (RCT) which reported that orbital radiotherapy was as effective as oral prednisolone[7]. However, the same authors subsequently found that radiotherapy was no better than placebo (except in a subgroup of patients with motility impairment) [14], and a recent trial from the Mayo Clinic in the USA also could not demonstrate a beneficial therapeutic effect [15]. As a result, many clinicians, particularly in North America, have abandoned its use.

The Mayo study has been widely criticised [16]and European groups argue that the balance of evidence remains in favour of radiotherapy [17]. In support of this, the most recent randomised control trial (in patients with relatively mild disease) found that radiotherapy was better than placebo when outcome was assessed by clinical measures [18]. However, this was not associated with quality-of-life or health economic gain, and the role of radiotherapy as monotherapy for TED remains the subject of heated debate [19-28].

Older RCTs have shown that radiotherapy is more effective in combination with steroids than when either agent is used alone [29,30]. However, this distinction between monotherapy and combination therapy is rarely highlighted, and the confusion which has resulted from the inconsistent recent evidence has caused many ophthalmologists to discontinue using radiotherapy as part of a combined treatment regime. Consequently there is a pressing need for a definitive study to investigate radiotherapy's role *in combination *with steroids.

The data supporting the use of ciclosporin combined with steroids [6,11], and radiotherapy combined with steroids [30,31] are not surprising given current knowledge about the benefits of combination therapies in other autoimmune ocular and systemic conditions [32,33]. Manipulation of an autoimmune response is more effective when more than one mechanism of immunosuppression is used, and this can be especially so early in the disease [32]. Combined drug treatments can also be continued long-term, preventing the disease reactivation which is commonly seen at the end of typical short-term low dose steroid monotherapy regimes, and enabling the duration of an individual's treatment to be tailored to the length of the active phase of their disease.

Azathioprine is a low-cost second-line immunosuppressive agent which is widely used in the management of other autoimmune conditions. It is better tolerated than ciclosporin [34] and does not cause the renal toxicity and cardiovascular side-effects [35] (including hypertension and hypercholesterolaemia) associated with ciclosporin. However, it can cause bone marrow suppression [36] and hepatotoxicity [37]. This risk has been greatly reduced with the advent of a laboratory assay for the enzyme thiopurine methyltransferase (TPMT), which regulates a key step in azathioprine metabolism. Now individuals with low TPMT activity, who would otherwise be at particular risk of azathioprine toxicity, can be identified and their treatment modified or withheld [38]. Hence, azathioprine has safety, tolerability and cost advantages over ciclosporin.

Retrospective data suggests that azathioprine, when used in combination with oral prednisolone and radiotherapy, can significantly reduce the long-term severity of TED and the need for rehabilitative surgery [39,40]. However, the use of azathioprine for TED remains highly controversial; in part because it has previously been proven ineffective as monotherapy [41]. As with radiotherapy, there is no clear evidence base and clinical practice consequently varies widely.

The current trial is designed to investigate whether (i) radiotherapy and (ii) azathioprine are effective *when used in combination with steroids *for the treatment of *active *TED. It will also provide pilot data on the possible benefits of triple therapy (steroids, azathioprine and radiotherapy). Particular attention in the trial design has been given to (a) establishing robust placebo controls and masking protocols which are effective and safe for both radiotherapy and the systemic administration of an antiproliferative drug; (b) constructing effective inclusion and exclusion criteria to select for *active *disease; and (c) selecting pragmatic outcome measures. Health economic and carefully designed quality of life/disfigurement analyses also accompany the trial.

## Methods/design

### Trial design

**Figure 1:**
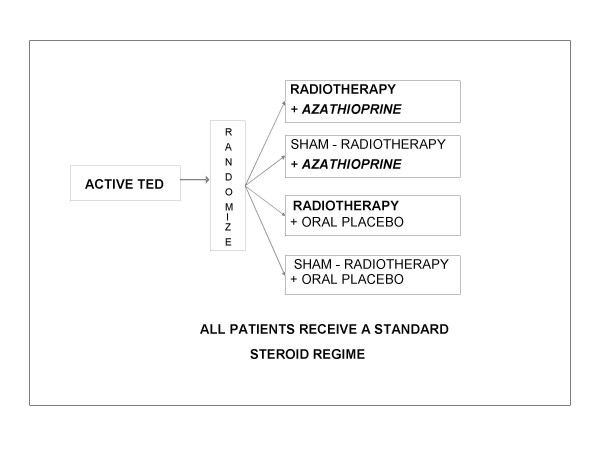
Trial Design.

Factorial design, double-masked, randomised controlled trial. Study recruits receive either azathioprine or placebo *plus *either radiotherapy or sham-radiotherapy, *in combination with *a standard oral prednisolone regime (Figure 1).

### Objectives

#### Primary

To test the hypotheses that in patients being treated with prednisolone for active TED:

1. Radiotherapy (compared with placebo) induces early remission and reduces long-term disease severity.

2. Azathioprine (compared with placebo) reduces long-term disease severity.

#### Secondary

1. To test the hypotheses that in patients being treated with prednisolone for active TED, radiotherapy and azathioprine improve health-related quality of life, and are cost-effective.

2. To validate the use of the GO-QoL (a new TED specific quality of life score) in the UK population.

3. To improve understanding of the extent and type of psychosocial distress experienced by TED patients.

4. To conduct an economic evaluation of the cost of TED and its treatment to patients, the National Health Service (NHS) and Society.

5. To report the safety and tolerability of radiotherapy and azathioprine in the study cohort.

### Eligibility

#### Inclusion criteria

• Clinical Activity Score (Table 1) ≥ 4 (worst eye) OR ≥ 2 (worst eye) with a history of proptosis or motility restriction of less than 6 months duration.

• Past or present history of abnormal Thyroid Gland Function OR a clinical diagnosis of TED made and confirmed by ≥ 2 muscle involvement on Computed Tomography (CT) or Magnetic Resonance Imaging (MRI) scan.

**Table 1:** Clinical Activity Score

**PAIN**	Pain on eye movement in the last 4 weeks
	Pain or oppressive feeling on or behind globe in the last 4 weeks
**REDNESS**	Conjunctival redness
	Eyelid redness
**SWELLING**	Chemosis
	Swollen caruncle
	Eyelid oedema
	* Increasing proptosis of ≥ 2 mm during a period of 1–3 months
**IMPAIRED FUNCTION**	* Decrease in eye movement in any direction of ≥ 8° during a period of 1–3 months
	* Decrease in visual acuity of ≥ 1 line on the Snellen chart (using a pinhole) during a period of 1–3 months

1 point for each feature present.

*Criteria only counted at follow-up visits.

Maximum Clinical Activity Score is 7 at enrolment visit and 10 at follow-up visits.

##### Definitions

• Proptosis: *Either *subjective unilateral proptosis confirmed by asymmetry in exophthalmometry of ≥ 2 mm *or *subjective bilateral proptosis.

• Motility restriction: Intermittent, inconstant or constant diplopia [42].

#### Exclusion criteria

• Age < 20 or > 75 yrs

• Dysthyroid optic neuropathy

• No proptosis or motility restriction (see above for definitions)

• Use of radioiodine within the last 3 months

• Previous orbital irradiation

• Pre-existing glaucoma with a characteristic optic disc appearance and associated visual field defect

• Pre-existing Diabetes Mellitus (not simply steroid induced disease from recent therapy)

• Previous adverse event associated with, or contraindication to, either prednisolone or azathioprine

• Within 6/12 of pregnancy, women planning pregnancy

• Lactation

• Haemoglobin Concentration > 1 g/dl below the local laboratory's reference range

• Platelet Count < 130 × 10^9^/L

• White Cell Count below the local laboratory's reference range

• Lymphocyte Count < 0.8 × 10^9^/L

• Abnormal Thiopurine Methyltransferase (TPMT) activity

• Abnormal renal function (estimated Glomerular Filtration Rate (eGFR) < 60 ml/min/1.73 m^2^)

• Abnormal liver function, specifically: bilirubin, alanine aminotransferase or alkaline phosphatase concentrations > 1.5× above the local laboratory's reference range

• Malignant or pre-malignant (dysplastic) condition within the past 5 years

• Previous tuberculosis

• Shingles within the past 3/12

• HIV/AIDS

• Concurrent use of other immunosuppressive agents or allopurinol

• Live vaccines within the past 3 months

All. patients referred to the Trial Centres with TED are screened, and those meeting the eligibility criteria are invited to enrol. The study is conducted according to ICH-GCP (International Conference on Harmonisation for Good Clinical Practice in clinical research), as set out in the European Union Clinical Trials Directive (2001) and associated UK Regulations (2004), which adhere to the principles of the Helsinki Declaration. At the first trial visit informed written consent is obtained and baseline characteristics measured, including completion of a quality of life questionnaire. Recruits then receive a standard course of high dose tapering prednisolone (Table 2 &3). If they *either *have a < 6 month history of TED (defined as time since first symptom) *or *report improvement in any item of the Clinical Activity Score (CAS) 2 weeks after starting the trial prednisolone regime, they are considered to have 'active' disease and are randomised at the second trial visit (Table 4).

**Table 2:** Prednisolone Treatment

**Time after enrolment**	**Prednisolone dose (mg/day)**
1–3 days	80
4–7 days	60
1–2 weeks	40
3–6 weeks	30
7–10 weeks	20
11–14 weeks	15
15–18 weeks	10
19–20 weeks	7.5
21–22 weeks	5
23–24 week	2.5

**Table 3:** Other Standardised Trial Treatments

Bisphosphonates	Risedronate sodium or alendronic acid
Proton pump inhibitors	Lansoprazole or omeprazole
Lubricants	Saline minims when required (no non-steroidal anti-inflammatory drops)

**Table 4:** Trial timeline

**Week**	**-2**	**0**	**1–3**	**4**	**5–7**	**13**	**20**	**24**	**28**	**36**	**44**	**48**
**Orthoptic assessment**	✓	✓		✓		✓		✓		✓		✓
**Clinical assessment**	✓	✓		✓		✓		✓		✓		✓
**Blood tests**	✓	✓	✓	✓		✓	✓	✓	✓	✓	✓	✓
**Quality of life questionnaires**	✓					✓						✓
**Prednisolone**	start							stop				
**Azathioprine/Placebo**		start										
**Radiotherapy/Sham-radiotherapy**					✓							

Week -2 = enrolment; Week 0 = randomisation

### Interventions

#### Radiotherapy

A dose of 20 Gy is administered to the retrobulbar orbit in 12 fractions over 2 to 3 weeks. The protocols used for treatment planning, verification, field arrangement and patient immobilisation vary according to the usual practice of each Trial Radiotherapy Centre.

In Bristol (Bristol Oncology Centre), patients are marked up on the treatment machine (a 6-MV linear accelerator) and immobilised with tape across their forehead. Isodose plans are not produced as a routine, but verification is carried out with Electronic Portal Imaging Devices (EPIDs) on days 1 and 2. Lasers are used to orientate the patient and radiation is delivered to 4 cm × 4 cm lateral opposed fields, using asymmetric jaws to eliminate divergence towards the contra-lateral lens.

In London (St Bartholomew's Hospital) and Manchester (Christie Hospital), all patients have an orbital CT scan prior to treatment as well as a dosimetric plan. Patients are immobilised with a full head shell, which is individually made for each trial subject, and radiation is delivered to 5 cm × 5 cm lateral opposed fields using a 6-MV linear accelerator. Thermoluminescence dosimetry (TLD) is then used for both eyes during the first treatment to check the lens dose.

#### Azathioprine

Treatment dose varies between 100 mg and 200 mg daily (dispensed as 50 mg tablets), depending on body weight (Table 5).

**Table 5:** Azathioprine & Placebo doses

**BODY MASS (KG)**	**AZATHIOPRINE STARTING DOSE (PER DAY)**	**NUMBER OF AZATHIOPRINE 50 MG TABLETS (PER DAY)**	**NUMBER OF CORRESPONDING PLACEBO TABLETS (PER DAY)**
< 50	100 mg	2	2
50 – 79	150 mg	3	3
≥ 80	200 mg	4	4

### Masking

Trial participants, the clinical investigators responsible for patient assessments, and data analysts are masked to treatment allocation. Masking success is evaluated at the end of the study with a questionnaire asking participants and investigators to state whether they think they know which treatments they received, and if so why.

### Placebo treatments

#### Sham – radiotherapy

In Bristol, treatment is planned as for active therapy, which does not involve radiation. However, in London and Manchester, patients having active treatment are exposed to radiation during both their CT scan and dosimetric planning. To avoid this in the sham-treatment group, sham-CT scans and a generic dosimetric plan with zero downloaded monitor units (ie no radiation exposure) are used.

Radiotherapy machines make a loud buzzing noise when switched on and this is the only indicator patients have that they are receiving treatment. In order to mimic this for patients allocated to receive sham-radiotherapy, bespoke noise-emitting devices (NEDs) which make a similar sound to that which accompanies normal treatment have been constructed, and are positioned above the gantry of the machines in Bristol, London and Manchester. The NEDs are activated from the radiotherapy suite's control room for the same duration of time that treatment would usually be administered. Hence, patients allocated to the sham-radiotherapy arm of the study have the same planning and treatment experience, but receive no radiation at all.

#### Placebo azathioprine tablets

These are made for the trial by St Thomas' Hospital Pharmacy Manufacturing Unit in accordance with Good Manufacturing Practice and exactly match the appearance of the active azathioprine tablets, which are removed from their blister-packs and re-packaged in the same bottles as the placebos. Both active and placebo drugs are then labelled as trial-specific Investigational Medicinal Products (IMPs) and distributed to the trial centres.

Placebo azathioprine tablets are also dispensed according to body weight, so patients in the placebo group take the same number of tablets per day as they would if receiving active treatment. (Table 5).

### Additional measures to maintain masking

#### Sham-radiotherapy

The trial radiographers are by necessity aware of each patient's treatment allocation. Their contact with trial recruits is therefore the time at which patients are at greatest risk of being un-masked. In order to minimise this risk, the number of radiographers involved in the administration of radiotherapy to trial subjects has been limited as much as possible. These individuals have all received specific training about the study and are very aware of the importance of maintaining masking, which they achieve by acting out their well-practiced treatment set-up and delivery protocols in a standard fashion to all patients regardless of whether they receive sham or active therapy. The clinical oncologists who consult with patients before and during treatment are not informed of treatment allocation, and the contents of patients' hospital records are specially adapted to keep this information concealed until they complete the study.

The radiotherapy departments involved in the trial have also examined their local facilities to ensure that other potential indicators of treatment allocation, such as the activation of warning lights outside radiotherapy suites when radiotherapy machines are being used, are identified and addressed (for example, taking care to sit patients and relatives out of sight of the warning lights).

#### Placebo azathioprine tablets

**Figure 2:**
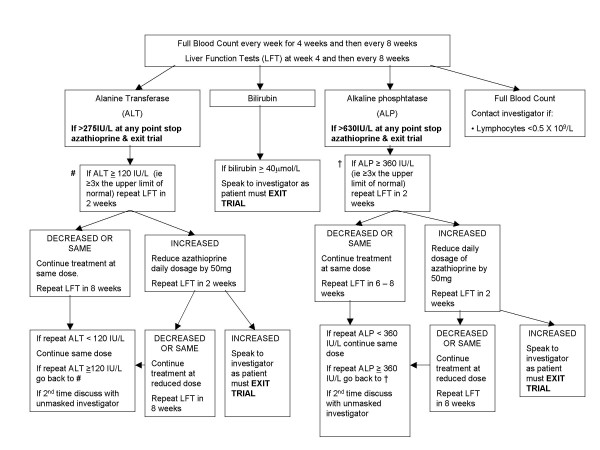
Azathioprine Dose Adjustment/Blood Test Algorithm.

All trial recruits, whether allocated to the azathioprine or placebo arms of the trial, will have regular blood tests to monitor for the potential adverse effects of azathioprine. These results are monitored by an un-masked trial co-ordinator, who arranges for trial subjects to be recalled if abnormalities are revealed and adjusts their treatment in accordance with a standard algorithm (Figure 2).

In order to maintain masking about 5% of the placebo treated group are also being randomly recalled for identical drug 'dose' adjustment, and repeat blood tests. The un-masked trial co-ordinator is never in direct contact with trial recruits, and the instructions issued to the clinical investigators to recall patients and adjust their treatment is standard, regardless of whether they are receiving active or placebo tablets. Hence, safety is ensured for patients on azathioprine, and treatment allocation is not revealed to the trial subjects or investigators.

If the un-masked trial co-ordinator has a query about a blood test result which is not adequately addressed by the standard treatment algorithm (Figure 2), they can reveal a patient's treatment allocation and ask for advice from one of the clinical investigators at another Trial Centre. This clause provides a safety net should an unforeseen clinical scenario arise, and should not introduce bias as the patient and the clinical investigator responsible for their assessment remains masked.

### Allocation of trial interventions

Patients who are eligible to continue in the study at the second trial visit (after confirmation of active disease by response to steroids or short disease duration – see above) are allocated to treatment groups by minimisation. This is a dynamic process which reduces the imbalance between trial arms with respect to a range of predefined prognostic variables, and a randomisation schedule is therefore not drawn up in advance. Instead, a form categorising each study recruit according to the minimisation criteria set out in Table 6 is returned to the randomisation service at Moorfields Eye Hospital on enrolment, giving them time to determine the patient's treatment allocation should they be eligible to proceed in the study. The trial pharmacists at each Trial Centre then phone the randomisation service on receipt of a trial IMP prescription at the patient's second visit, and are notified whether they are to dispense azathioprine or placebo. This is confirmed by email, which also states the patient's radiotherapy group – a copy of which is sent to the Trial Radiotherapy Centre radiographers and the un-masked trial co-ordinator.

**Table 6:** Minimisation Criteria

**Minimisation criteria**	**Categories**
Smoker at time of TED diagnosis	Yes/No
Previous Steroid use*	Yes/No
Gender	Male/Female
Disease severity	TES < 22/TES ≥ 22
Study Centre	MEH/BEH/WEH/UCLH/MREH
Disease duration	< 6 months/≥ 6 months
Age	< 60 years old/≥ 60 years old
Disease activity	CAS 2–3/4–5/6–7
Thyroid status on enrolment	Hypothyroid/Euthyroid/Hyperthyroid

* Defined as ≥ 20 mg of prednisolone for ≥ 1 month in the previous 6 months

TES: Total Eye Score; MEH: Moorfields Eye Hospital; BEH: Bristol Eye Hospital; WEH: Western Eye Hospital; UCLH: University College London Hospital; MREH: Manchester Royal Eye Hospital; CAS: Clinical Activity Score.

### Further measures to reduce bias

#### Standard Patient Assessments

An 'atlas' of standard photographs is being used for reference in clinical assessments to reduce inter-observer differences in the measurement of disease activity and severity [43].

### Management of other factors which have the potential to influence TED

#### Endocrine Management

**Figure 3:**
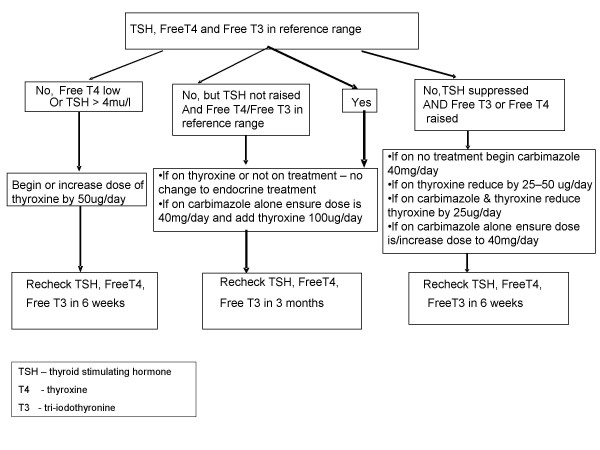
Endocrine Treatment Protocol.

The protocol for endocrine treatment is outlined in Figure 3. This will be administered in liaison with the patient's endocrinologist, or if the patient is not under the care of an endocrinologist when enrolled, by the trial investigators. The trial endocrinologist (CMD) will mediate if a patient's endocrinologist does not adhere to the trial protocol, and all protocol deviations will be reported at the end of the study.

#### Smoking

All trial subjects are advised to stop smoking and changes in smoking habits are recorded.

### Follow-up

The timing and content of trial assessments are illustrated in Table 4.

In addition to completing quality of life questionnaires, a subset of patients will also be selected to participate in a semi-structured interview to explore their individual concerns, strengths and difficulties in social functioning, coping strategies, levels of perceived support and the personal financial consequences of their disease. Interviews will be tape recorded and subsequently transcribed, after which the tapes will be erased (transcriptions will also be destroyed at the end of the study).

At the second study visit all patients are issued with a trial diary (see Additional file 1). This records a wide variety of information relevant to their treatment costs, including use of primary and secondary healthcare, prescriptions, travel costs, over-the-counter medications, cosmetics and loss of earnings.

### Outcome measures

#### Primary

**Figure 4:**
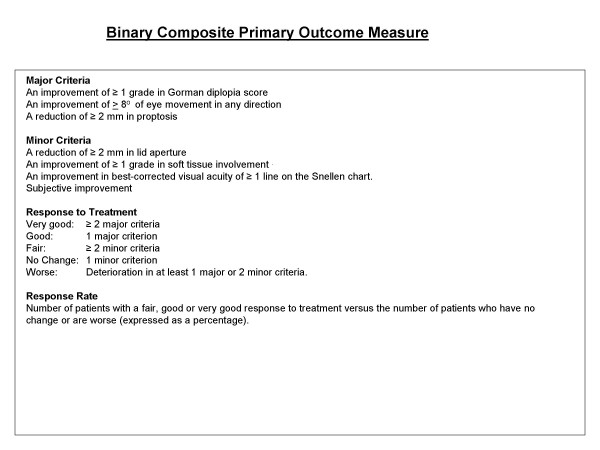
Binary Composite Primary Outcome Measure.

*1. Binary composite outcome score*:

Treatment success and failure will be defined at study completion (1 year) using a system of major and minor criteria modified from others [8,14,18] (Figure 4).

*2. Ophthalmopathy index *[44]:

Treatment response at study completion (1 year) will also be quantified using the Ophthalmopathy Index (OI) as a continuous measure of disease *severity*.

#### Co-primary

*1. Clinical activity score *[45,46]:

Short-term response to treatment (3 months) will be evaluated using the Clinical Activity Score (CAS, Table 1) as a continuous measure of disease *activity*.

#### Secondary

1. *Clinical measures:*

Total eye score (TES) [6].

2. *Psychosocial measures *[see Additional file 2]:

- Hospital Anxiety and Depression Scale (HADS) score [47]

- Derriford Appearance Scale-Short Form score [48,49]

- Graves' Ophthalmopathy Quality of Life (GO-QoL) score [50,51]

- WHOQoL-Brief Quality of Life Assessment Score [52]

- Open-ended responses to interview questions

3. *Health economic measures *[see Additional file 1]:

The cost of TED and its treatment will be measured from the perspective of the NHS, the patient and society.

#### Adverse events

Adverse events will be documented and serious adverse events will be reported to the trial sponsor and to the Medicines and Healthcare Products Regulatory Agency (MHRA). Specific enquiries regarding known side-effects of the active interventions in the trial (as documented below) will be made at each visit and documented. Clinical examination will include monitoring for adverse events.

*Prednisolone *– gastric irritation/ulceration, weight gain, sleep disturbance, mood

disturbance, increased susceptibility to infection, increase in blood pressure, steroid induced diabetes.

*Azathioprine *– malaise, nausea, vomiting, diarrhoea, rash, myalgia, hair loss, increased susceptibility to infection. Regular blood tests will be carried out on all patients to monitor for bone marrow suppression and liver impairment.

*Radiotherapy *– localized redness of skin, cataracts, radiation retinopathy.

As this is a double-masked trial all adverse events will be documented and reviewed by the data monitoring committee who may be unmasked to treatment allocation.

### Trial size

#### Number of patients

A two group continuity corrected chi-squared test with a 0.050 two-sided significance level will have 80% power to detect the difference between a success rate in the placebo arm of 60% and success rate in the treated arm of 87% (odds ratio of 4.462) when the sample size in each group is 48. It is therefore necessary for there to be 96 subjects with complete final data sets to be equally distributed to the 4 treatment groups.

The power for continuous outcome measures is more favourable and a total sample size of 100 patients will yield 92% power to detect a difference of 2.7 in the OI with two-sided 5% alpha, assuming a standard deviation of 3.3.

CAS is not considered as important as the primary outcomes in terms of influencing future clinical practice. However it is of greater interest and importance than the secondary outcome variables, and as such it has been selected as a coprimary endpoint and the power of the trial to detect a clinically important difference is presented here. Assuming a standard deviation of between 1.2 and 2.0, a total sample of 100 patients will yield between 71% and 99% power to detect a difference of 1 point with 5% two-sided alpha.

The above power calculations are based on comparisons 'at the margins' of the factorial design (ie, comparing all azathioprine treated patients with placebo treated patients, and all radiotherapy treated patients with sham-radiotherapy treated patients). There will be low power for comparisons between individual cells of the factorial design (eg azathioprine and sham-radiotherapy vs. placebo and radiotherapy), although these analyses will still be conducted to generate pilot data on the comparative efficacy of the 4 combination regimes under investigation.

#### Compliance and loss to follow-up

We predict that 33% of eligible patients will choose not to take part in the study [14], 11% will be excluded because of TPMT inactivity [53], 10% of the azathioprine treated group will be intolerant of therapy or suffer an adverse event necessitating withdrawal from the trial [54], and 5% will drop-out for other reasons.

### Statistical analyses

Data analysis will proceed according to CONSORT guidelines for randomised controlled trials. The first stage of the analysis will be to use descriptive statistics to describe the group of individuals recruited to the trial in relation to those eligible, and to investigate comparability of the trial arms at baseline. The primary intention-to-treat analyses will comprise comparisons between radiotherapy versus sham-radiotherapy, and azathioprine versus placebo, for each of the two primary outcomes at 12 months follow up. The comparisons will be made using appropriate (that is, logistic or linear) multivariable regression models, adjusting for minimisation variables, the factorial design, and the value of the outcome variable at baseline. Full attention will be paid to the estimates and the confidence intervals for these comparisons as well as the p-values. Secondary analyses will then be conducted using regression models with further adjustment for any prognostic factors that exhibit marked imbalance at baseline. Patients who have no outcome data for the primary analyses will have data imputed using last observation carried forward. Patients who have become non-compliant for any reason will still be invited to produce outcome data such that they can be included in the intention to treat analyses. The assumptions required for the regression models will be investigated using appropriate diagnostic plots, and actions such as transformation of continuous outcome variables taken as necessary.

The co-primary outcome CAS at 3 months follow up, and all secondary outcomes, will be analysed in the same way, using appropriate (linear or logistic) regression models depending on the nature of the outcome measure. Bonferroni corrections for multiple testing will be considered for the secondary outcomes.

Other secondary analyses will involve: (a) investigation of any interaction between the two interventions for each of the two primary outcomes; (b) pre-planned sub-group analyses to ascertain any differential effects of the interventions according to steroid use versus no steroid use in the six months prior to enrolment. These secondary analyses are readily performed as extensions to the multivariable regression models described above, by simply introducing the appropriate interaction terms. However, the precision of the estimates of interaction is very likely to be too poor, and high p-values will most likely reflect low power and so cannot be taken as evidence for no interactions.

#### Economic evaluation

Costs will be related to the percentage of patients responding to treatment. Incremental cost effectiveness ratios will be formed, which will estimate the extra cost per extra patient responding to (i) radiotherapy; and (ii) azathioprine 12 months after randomisation. A secondary analysis will estimate the cost-of-illness of TED from a societal perspective.

### Trial organisation

Trial Centres and Investigators are listed in Table 7. Additional trial centres may be added if recruitment is slower than anticipated.

**Table 7:** Trial Centres & Principal Investigators

**Trial sponsor**	University of Bristol
**Trial Centres and Principal Investigators**	

Bristol Eye Hospital and University of Bristol	Richard Lee and Colin Dayan
Moorfields Eye Hospital	Jimmy Uddin
University College Hospital	Fion Bremner, Steve Hurel
Western Eye Hospital	Jane Olver, Rajni Jain
Manchester Royal Eye Hospital	Anne Cook

**Radiotherapy Centres**	

Bristol Oncology Centre	Marjorie Tomlinson
St Bartholomew's Hospital	Nicholas Plowman
The Christie Hospital	Rao Gattamaneni

#### Trial Steering Committee

The Trial Steering Committee will monitor and supervise the trial and comment on any proposed major protocol amendments (Table 8).

**Table 8:** Trial management

**Core trial management committee**	
Richard Lee	Chief Investigator (Ophthalmology), University of Bristol and Bristol Eye Hospital, UK
Colin Dayan	Chief Investigator (Endocrinology), University of Bristol and Bristol Royal Infirmary, UK
Jimmy Uddin	Principal investigator (Ophthalmology), Moorfields Eye Hospital. London, UK
Rathie Rajendram	Co-investigator (Ophthalmology), Moorfields Eye Hospital, London, UK
Catey Bunce	Medical statistician, Moorfields Eye Hospital, London, UK

**Trial Steering Committee**	

Maarten P Mourits	Professor of Ophthalmology, Academic Medical Centre, Amsterdam, NL
John Lazarus	Professor of Endocrinology, University of Wales, Cardiff, UK
John Sparrow	Consultant Ophthalmologist, Bristol Eye Hospital, UK

**Data Monitoring Committee**	

John Forrester	Professor of Ophthalmology, University of Aberdeen and Aberdeen Royal Infirmary, UK
Gillian Adams	Consultant Ophthalmologist, Moorfields Eye Hospital, London, UK
Roberto Melotti	Senior statistician, North Bristol NHS Trust, Bristol, UK

#### Data Monitoring Committee

The data monitoring committee (DMC) includes an independent ophthalmologist with experience of thyroid eye disease and an independent statistician (Table 8).

No formal interim analysis is planned. The trial statistician will report to an independent DMC which will monitor the trial in all its respects. It will review data from the trial every 12 months, consider the findings from other relevant studies and if considered appropriate may conduct a masked interim analysis. The trial will only be terminated as a result of this analysis if the p value is smaller than 0.0001 and the DMC decides that this is the best course of action to take.

#### Study co-ordination

This study will be centrally co-ordinated by Moorfields Eye Hospital Clinical Trials Unit (CTU). As well as providing the telephone randomisation service the CTU will be responsible for data management.

### Trial documentation and data collection

The trial centres are provided with a Protocol, Standard Operating Procedures [see Additional file 3] (including standardised colour plates for patient assessments [43]), Source Documentation and Case Report Forms [see Additional file 4]. Adverse event forms are completed at each participant's visit. Serious adverse events will be reported to the University of Bristol (the Trial Sponsor) and to the Medicines and Healthcare products Regulatory Agency (MHRA).

### Ethics and competent authority review

Applications to UK Main and Local Research Ethics Committee (REC) have received favourable opinions and a Clinical Trials Authorisation has been issued by the MHRA.

### Publication policy

The results of this trial will be submitted for publication in peer-reviewed medical journals regardless of whether the outcome is in favour of the trial interventions. Authorship agreements have been signed by the investigators prior to the start of the study.

### Proposed trial time-table

#### Trial start

January 2006

#### Projected trial end

December 2011

#### Trial duration

6 Years

#### Duration of each patient's participation

12 months

## Discussion

This randomised controlled trial of azathioprine and radiotherapy (with prednisolone) will be the first to evaluate the role of these interventions as part of a long-term combined immunosuppressive treatment regime for TED. We hope to help resolve the debate which has arisen from recent monotherapy studies [16,55], and seek not only to establish whether the addition of azathioprine or radiotherapy confers benefit in patients treated with glucocorticoids, but also to test the principle of combination therapies in TED.

The use of a factorial design enables us to evaluate two interventions in a single study and is the most valid means of establishing whether the combination of two or more therapies achieves incremental benefits [56]. It also makes efficient use of patients, which is especially important because of the limited numbers of potential recruits with active TED [56,57]. However, in using a factorial design we are assuming (but will have insufficient power to test) that there is no interaction between trial interventions, as if a significant biological or statistical interaction between radiotherapy and azathioprine treatment exists, our calculation of their independent effects will be incorrect. The validity of the study design therefore rests on the judgement of the trial investigators and statistical advisors that the potential for an interaction between our interventions to have a 'clinically significant' effect on the interpretation of the trial data is very low (ie ≤ 5%). We believe this to be a reasonable assertion and consider that the risk of generating uninterpretable data is outweighed by the benefits of using a factorial design to assess combination treatments for an uncommon condition. Our small sample size will also limit our sensitivity to small differences in treatment efficacy, which is a compromise we accept as integral to the conduct of a pragmatic study.

In addition to the restricted number of trial subjects, there are many factors in the natural history and clinical evaluation of TED which pose challenges to the objective and scientific comparison of treatment outcomes. For example, the control of systemic thyroid dysfunction reduces disease severity, but delay in TED treatment until a 2 month period of euthyroidism has been maintained (as has been advocated by other investigators [7,9,14,18]) in order to prevent this confounding interpretation of treatment efficacy, potentially misses the opportunity to obtain maximal benefit from immunosuppression in the earliest, most active phase of the disease. A patient's smoking status, previous exposure to steroids and disease severity can also independently influence their response to treatment. Furthermore, measures of disease activity and severity are notoriously subjective [58,59], and although some outcome measures have been used more often than others in previous studies, there is no single standardised and robustly validated scoring system to use in the assessment of treatment efficacy.

We have sought to address the potential influence of multiple confounding factors through the use of a minimisation strategy to ensure their even distribution across trial arms (Table 6). The absence of validated outcomes is more problematic, particularly with regard to measures of disease severity, which need to incorporate a range of parameters to quantify both cosmetic deformity and visual dysfunction. The available continuous scoring systems [6,44] can be relatively insensitive to clinically significant treatment differences and depend on large sample sizes. Consequently, recent studies have tended to use binary composite outcome measures, weighted to detect improvement in a selected disease components, such as oculomotility [8,42,43,46]. However, no two studies have used the same criteria to define treatment success, and we have combined elements of each in order to generate our scoring system (Figure 4). There is greater consensus on the measurement of clinical activity and the CAS is an accepted standard which has been widely used in recent studies [8,14,15,18,60,61], however it remains limited by its subjectivity [58,59] and inability to predict response to steroids [45].

In order to ameliorate the problem of subjectivity in clinical assessments with consequent inter-observer variability we are using Dickinson et al.'s [43] reference atlas of colour plates and our standard operating procedures for patient examinations are based on those advocated by the European Group on Graves Ophthalmopathy (EUGOGO). In addition, we are seeking to lay the foundations for fully objective measurement of disease activity and severity through an ancillary Magnetic Resonance Imaging (MRI) study which is being conducted alongside the trial.

We have gone to great lengths to establish placebo controls for the trial interventions, in particular with sham-radiotherapy and decoy dose changes for patients allocated to receive placebo tablets. However, there is a risk that the common side-effects of azathioprine, such as lethargy and nausea, will unmask investigators and patients to their treatment allocation. This may be off-set by the overlap between the side-effect profiles of azathioprine and prednisolone and the success of our masking procedures will ultimately be evaluated by questionnaire when each patient completes the trial.

Referral patterns to the study centres dictate that it is impractical to restrict trial entry to patients who have not been previously exposed to steroids. Hence, all potential recruits who have sufficiently active disease to be eligible for trial entry will be enrolled regardless of their prior steroid exposure, and the potential this has to bias their subsequent immunoresponsiveness is accounted for in the minimisation criteria. In addition, the practicalities of day-case admission for intravenous (IV) prednisolone treatment have prevented the trial centres from adopting this as their usual practice, despite recent evidence in favour of IV administration [62], and we are consequently using oral prednisolone in the trial.

In summary, the CIRTED trial [63] seeks to utilise a high-quality, pragmatic trial design to evaluate combination immunotherapies for the treatment of TED and enhance the evidence base available to inform treatment decisions for both patients and health care professionals.

## Abbreviations

CAS: Clinical Activity Score; CT: Computed Tomography; CTU: Clinical Trials Unit; DMC: Data Monitoring Committee; EUGOGO: European Group on Graves Ophthalmopathy; GC: Glucocorticoids; GO-QoL: Graves Ophthalmopathy Quality of Life; HADS: Hospital Anxiety and Depression Scale; IMP: Investigational Medicinal Product; MHRA: Medicines and Healthcare products Regulatory Agency; MRI: Magnetic Resonance Imaging; NED: Noise-emitting device; NHS: National Health Service; OI: Ophthalmopathy Index; RCT: Randomised Control Trial; REC: Research Ethics Committee; TED: Thyroid Eye Disease; TES: Total Eye Score; TLD: Thermoluminescence Dosimetry; TPMT: Thiopurine Methyltransferase

## Competing interests

The author(s) declare that they have no competing interests.

## Authors' contributions

RR: Participated in development of the trial protocol. Coordinated the trial's set-up at Moorfields Eye Hospital and facilitated the set-up of the other study sites in London. Prepared the trial's standard operating procedures, study documentation and publicity. Drafted the manuscript.

RWJL: Conceived and designed the trial. Secured trial funding and coordinated its multi-centre management. Led the trial's set-up at Bristol Eye Hospital. Prepared the trial's standard operating procedures, study documentation and publicity. Drafted the manuscript.

MJP: Participated in development of the trial protocol.

GER: Contributed to trial design and participated in development of the trial protocol.

RJ and JMO: Led the trial's set-up and coordination at the Western Eye Hospital, London.

FB, SH: Led the trial's set-up and coordination at University College London Hospitals.

AC: Led the trial's set-up and coordination at Manchester Royal Eye Hospital.

RG: Led the set-up of trial radiotherapy at the Christie Hospital, Manchester.

MT: Responsible for trial radiotherapy at Bristol Oncology Centre.

NP: Led the trial's radiotherapy set-up at St Bartholomew's Hospital, London.

CB: Participated in development of the trial protocol. Set-up the trial's randomisation procedure. Coordinates statistical analyses.

SPH: Designed and coordinates the trial's health economic evaluation.

ADD: Helped to secure trial funding and facilitated its set-up at Bristol Eye Hospital.

NR, SJ and LK: Designed, implement and analyse the trial's psychosocial assessments.

OCM: Leads coordination at Moorfields Eye Hospital.

CMD: Conceived and designed the trial. Secured trial funding and coordinated its multi-centre management. Designed and coordinates the trial's endocrine management protocol. Drafted the manuscript.

JMU: Conceived and designed the trial. Secured trial funding and led its set-up at Moorfields Eye Hospital. Helped to draft the manuscript.

All authors read and approved the final manuscript.

## Supplementary Material

Additional file 1 Patient Diary: Resource use, Personal Costs and Health Record. A diary for patients to keep their appointments in and to document travel and other related expenses. Click here for file

Additional file 2 Quality of Life Questionnaires. Questionnaire to assess the patent's quality of life. This material uses the WHOQOL-UK and the assistance of the University of Bath and the World Health Organisation is acknowledged. Click here for file

Additional file 3 Standard Operating Procedures. Standard operating procedures for the combined immunosuppression & radiotherapy in thyroid eye disease (CIRTED) trial. This material is a modification of the Standard Operating Procedures advocated by the European Group on Graves Ophthalmopathy (EUGOGO), and utilises Dickinson & Perros's reference atlas of colour plates [43]. Click here for file

Additional file 4 Case Report Form. Case report form used in the combined immunosuppression & radiotherapy in thyroid eye disease (CIRTED) trial. Click here for file
