# Supplementary material for: Protocol for the combined immunosuppression & radiotherapy in thyroid eye disease (CIRTED) trial: A multi-centre, double-masked, factorial randomised controlled trial
Source: Trials. 2008 Jan 31;9:6. doi: 10.1186/1745-6215-9-6 (PMC2275219; doi:10.1186/1745-6215-9-6)
Supplement: Additional file 1 — Patient Diary: Resource use, Personal Costs and Health Record. A diary for patients to keep their appointments in and to document travel and other related expenses. [file 1745-6215-9-6-S1.pdf]

Moorfields Eye Hospital NHS
NHS Foundation Trust

CIRTED TRIAL

DIARY

University of BRISTOL

CIRTED Trial Diary v1.0; Sept 2005

CONTENTS

Introduction...1

Useful Contacts 3

Trial Medication Record – Trial Tablets & Prednisolone....5

Other Medication Record 21

Health Record 33

NHS / Trial Appointments..39

Eye Hospital Appointments...41

Radiotherapy Appointments..45

Endocrinology Appointments 49

General Practitioner (GP) Appointments........................53

Hospital Admissions 63

Other NHS Contacts (including Phone Calls)....................67

Use of Non- NHS Services 75

Thyroid Eye Disease (TED) Related Expenses..................87

Notes...97

Introduction

The main purpose of this diary is to record the costs you incur because of your Thyroid Eye Disease (TED). At present very little is understood about the financial implications of TED, to both patients and the health service, and we now have a unique opportunity to properly investigate this through the CIRTED trial. The cost of having an illness and its treatment can often be overlooked by the medical profession, and by completing this diary you will be providing information which we hope to use for the benefit of future TED sufferers.

To do this properly, we would like you to record all contact with health care services (NHS or non-NHS), medication use and new health problems which arise while you are participating in the trial – whether you think these are related to your TED or not. We consequently hope that this diary will serve as a useful organisational aid while you are in the study.

There are separate sections to record appointment times and to document your use of the trial medications.

We also wish to record your expenditure on TED related items (such as sunglasses and cosmetics)

Please take time to flick through the diary and familiarise yourself with its contents. Only complete the sections appropriate to you. We expect that you will only need to use a small fraction of the available space during your time in the study.

Your ‘trial identifier’ is ....

Please document this in the space provided at the bottom of each diary page you fill in.

Useful Contacts

- **www.cirted.org**
- *Your CIRTED trial doctor is*;

  ……………………………………………

  Pager:……………………………………

- *General Practitioner*

  Name:..

  Tel:...

  Other Details:..

  ....

  ....

Other Contacts:

- Name:...

  Tel:..

  Other Details:...

  ....

  ....

- Name:...

  Tel:..

  Other Details:...

  ....

  ....

Trial Medication Record – Trial Tablets & Prednisolone

Please complete this diary on a daily basis. Write the number of Trial Tablets (Azathioprine 50mg or Placebo) and the Prednisolone dose (eg ‘30mg’) that you have taken each day in the appropriate box, and also record the date (day / month / year) for the start of each week in the top left-hand box of each table. If you have forgotten to take your tablets please write in “0”, but remember to take your prescribed dose at the next scheduled time.

Week of: / /	Mon	Tues	Weds	Thurs	Fri	Sat	Sun
Number of Trial Tablets							
Prednisolone Dose							

Week of: / /	Mon	Tues	Weds	Thurs	Fri	Sat	Sun
Number of Trial Tablets							
Prednisolone Dose							

Week of: / /	Mon	Tues	Weds	Thurs	Fri	Sat	Sun
Number of Trial Tablets							
Prednisolone Dose							

Week of: / /	Mon	Tues	Weds	Thurs	Fri	Sat	Sun
Number of Trial Tablets							
Prednisolone Dose							

Week of: / /	Mon	Tues	Weds	Thurs	Fri	Sat	Sun
Number of Trial Tablets							
Prednisolone Dose							

Week of: / /	Mon	Tues	Weds	Thurs	Fri	Sat	Sun
Number of Trial Tablets							
Prednisolone Dose							

Week of: / /	Mon	Tues	Weds	Thurs	Fri	Sat	Sun
Number of Trial Tablets							
Prednisolone Dose							

Week of: / /	Mon	Tues	Weds	Thurs	Fri	Sat	Sun
Number of Trial Tablets							
Prednisolone Dose							

Week of: / /	Mon	Tues	Weds	Thurs	Fri	Sat	Sun
Number of Trial Tablets							
Prednisolone Dose							

Week of: / /	Mon	Tues	Weds	Thurs	Fri	Sat	Sun
Number of Trial Tablets							
Prednisolone Dose							

Week of: / /	Mon	Tues	Weds	Thurs	Fri	Sat	Sun
Number of Trial Tablets							
Prednisolone Dose							

Week of: / /	Mon	Tues	Weds	Thurs	Fri	Sat	Sun
Number of Trial Tablets							
Prednisolone Dose							

Week of: / /	Mon	Tues	Weds	Thurs	Fri	Sat	Sun
Number of Trial Tablets							
Prednisolone Dose							

Week of: / /	Mon	Tues	Weds	Thurs	Fri	Sat	Sun
Number of Trial Tablets							
Prednisolone Dose							

Week of: / /	Mon	Tues	Weds	Thurs	Fri	Sat	Sun
Number of Trial Tablets							
Prednisolone Dose							

Week of: / /	Mon	Tues	Weds	Thurs	Fri	Sat	Sun
Number of Trial Tablets							
Prednisolone Dose							

Week of: / /	Mon	Tues	Weds	Thurs	Fri	Sat	Sun
Number of Trial Tablets							
Prednisolone Dose							

Week of: / /	Mon	Tues	Weds	Thurs	Fri	Sat	Sun
Number of Trial Tablets							
Prednisolone Dose							

Week of: / /	Mon	Tues	Weds	Thurs	Fri	Sat	Sun
Number of Trial Tablets							
Prednisolone Dose							

Week of: / /	Mon	Tues	Weds	Thurs	Fri	Sat	Sun
Number of Trial Tablets							
Prednisolone Dose							

Week of: / /	Mon	Tues	Weds	Thurs	Fri	Sat	Sun
Number of Trial Tablets							
Prednisolone Dose							

Week of: / /	Mon	Tues	Weds	Thurs	Fri	Sat	Sun
Number of Trial Tablets							
Prednisolone Dose							

Week of: / /	Mon	Tues	Weds	Thurs	Fri	Sat	Sun
Number of Trial Tablets							
Prednisolone Dose							

Week of: / /	Mon	Tues	Weds	Thurs	Fri	Sat	Sun
Number of Trial Tablets							

Week of: / /	Mon	Tues	Weds	Thurs	Fri	Sat	Sun
Number of Trial Tablets							

Week of: / /	Mon	Tues	Weds	Thurs	Fri	Sat	Sun
Number of Trial Tablets							

Week of: / /	Mon	Tues	Weds	Thurs	Fri	Sat	Sun
Number of Trial Tablets							

Week of: / /	Mon	Tues	Weds	Thurs	Fri	Sat	Sun
Number of Trial Tablets							

Week of: / /	Mon	Tues	Weds	Thurs	Fri	Sat	Sun
Number of Trial Tablets							

Week of: / /	Mon	Tues	Weds	Thurs	Fri	Sat	Sun
Number of Trial Tablets							

Week of: / /	Mon	Tues	Weds	Thurs	Fri	Sat	Sun
Number of Trial Tablets							

Week of: / /	Mon	Tues	Weds	Thurs	Fri	Sat	Sun
Number of Trial Tablets							

Week of: / /	Mon	Tues	Weds	Thurs	Fri	Sat	Sun
Number of Trial Tablets							

Week of: / /	Mon	Tues	Weds	Thurs	Fri	Sat	Sun
Number of Trial Tablets							

Week of: / /	Mon	Tues	Weds	Thurs	Fri	Sat	Sun
Number of Trial Tablets							

Week of: / /	Mon	Tues	Weds	Thurs	Fri	Sat	Sun
Number of Trial Tablets							

Week of: / /	Mon	Tues	Weds	Thurs	Fri	Sat	Sun
Number of Trial Tablets							

Week of: / /	Mon	Tues	Weds	Thurs	Fri	Sat	Sun
Number of Trial Tablets							

Week of: / /	Mon	Tues	Weds	Thurs	Fri	Sat	Sun
Number of Trial Tablets							

Week of: / /	Mon	Tues	Weds	Thurs	Fri	Sat	Sun
Number of Trial Tablets							

Week of: / /	Mon	Tues	Weds	Thurs	Fri	Sat	Sun
Number of Trial Tablets							

Week of: / /	Mon	Tues	Weds	Thurs	Fri	Sat	Sun
Number of Trial Tablets							

Week of: / /	Mon	Tues	Weds	Thurs	Fri	Sat	Sun
Number of Trial Tablets							

Week of: / /	Mon	Tues	Weds	Thurs	Fri	Sat	Sun
Number of Trial Tablets							

Week of: / /	Mon	Tues	Weds	Thurs	Fri	Sat	Sun
Number of Trial Tablets							

Week of: / /	Mon	Tues	Weds	Thurs	Fri	Sat	Sun
Number of Trial Tablets							

Week of: / /	Mon	Tues	Weds	Thurs	Fri	Sat	Sun
Number of Trial Tablets							

Week of: / /	Mon	Tues	Weds	Thurs	Fri	Sat	Sun
Number of Trial Tablets							

Week of: / /	Mon	Tues	Weds	Thurs	Fri	Sat	Sun
Number of Trial Tablets							

Week of: / /	Mon	Tues	Weds	Thurs	Fri	Sat	Sun
Number of Trial Tablets							

Week of: / /	Mon	Tues	Weds	Thurs	Fri	Sat	Sun
Number of Trial Tablets							

Other Medication Record

Please write down the name and dose of all tablets and medications you take (either prescribed by a doctor, or purchased over-the-counter) ***other*** than treatments we give you at the Eye Hospital. If you are taking these other medications for your thyroid eye disease (eg pain killers for discomfort around the eyes), or to counteract the side-effects of the treatment you have been given for your Thyroid Eye Disease (such as indigestion associated with steroid use), we would also like to record their cost.

Medication (Name)	Dose (eg '5mg')	Frequency (eg 2 x / day)	Date Started	Date Stopped

If associated with TED or its treatment please record the cost (or prescription charge) of each purchase

£	.	.	.	.	.	.	.	.	.
	.	.	.	.	.	.	.	.	.

Medication (Name)	Dose (eg '5mg')	Frequency (eg 2 x / day)	Date Started	Date Stopped

If associated with TED or its treatment please record the cost (or prescription charge) of each purchase

£									
	.	.	.	.	.	.	.	.	.
	.	.	.	.	.	.	.	.	.

Medication (Name)	Dose (eg '5mg')	Frequency (eg 2 x / day)	Date Started	Date Stopped

If associated with TED or its treatment please record the cost (or prescription charge) of each purchase

£									
	.	.	.	.	.	.	.	.	.
	.	.	.	.	.	.	.	.	.

Medication (Name)	Dose (eg ‘5mg’)	Frequency (eg 2 x / day)	Date Started	Date Stopped

If associated with TED or its treatment please record the cost (or prescription charge) of each purchase

£									
	.	.	.	.	.	.	.	.	.
	.	.	.	.	.	.	.	.	.

Medication (Name)	Dose (eg ‘5mg’)	Frequency (eg 2 x / day)	Date Started	Date Stopped

If associated with TED or its treatment please record the cost (or prescription charge) of each purchase

£									
	.	.	.	.	.	.	.	.	.
	.	.	.	.	.	.	.	.	.

Medication (Name)	Dose (eg ‘5mg’)	Frequency (eg 2 x / day)	Date Started	Date Stopped

If associated with TED or its treatment please record the cost (or prescription charge) of each purchase

£	.	.	.	.	.	.	.	.	.
	.	.	.	.	.	.	.	.	.

Medication (Name)	Dose (eg ‘5mg’)	Frequency (eg 2 x / day)	Date Started	Date Stopped

If associated with TED or its treatment please record the cost (or prescription charge) of each purchase

£	.	.	.	.	.	.	.	.	.
	.	.	.	.	.	.	.	.	.

Medication (Name)	Dose (eg ‘5mg’)	Frequency (eg 2 x / day)	Date Started	Date Stopped

If associated with TED or its treatment please record the cost (or prescription charge) of each purchase

£								
.	.	.	.	.	.	.	.	.
.	.	.	.	.	.	.	.	.

Medication (Name)	Dose (eg ‘5mg’)	Frequency (eg 2 x / day)	Date Started	Date Stopped

If associated with TED or its treatment please record the cost (or prescription charge) of each purchase

£								
.	.	.	.	.	.	.	.	.
.	.	.	.	.	.	.	.	.

Medication (Name)	Dose (eg ‘5mg’)	Frequency (eg 2 x / day)	Date Started	Date Stopped

If associated with TED or its treatment please record the cost (or prescription charge) of each purchase

£									
	.	.	.	.	.	.	.	.	.
	.	.	.	.	.	.	.	.	.

Medication (Name)	Dose (eg ‘5mg’)	Frequency (eg 2 x / day)	Date Started	Date Stopped

If associated with TED or its treatment please record the cost (or prescription charge) of each purchase

£									
	.	.	.	.	.	.	.	.	.
	.	.	.	.	.	.	.	.	.

Medication (Name)	Dose (eg ‘5mg’)	Frequency (eg 2 x / day)	Date Started	Date Stopped

If associated with TED or its treatment please record the cost (or prescription charge) of each purchase

£	.	.	.	.	.	.	.	.	.
	.	.	.	.	.	.	.	.	.

Medication (Name)	Dose (eg ‘5mg’)	Frequency (eg 2 x / day)	Date Started	Date Stopped

If associated with TED or its treatment please record the cost (or prescription charge) of each purchase

£	.	.	.	.	.	.	.	.	.
	.	.	.	.	.	.	.	.	.

Medication (Name)	Dose (eg '5mg')	Frequency (eg 2 x / day)	Date Started	Date Stopped

If associated with TED or its treatment please record the cost (or prescription charge) of each purchase

£	.	.	.	.	.	.	.	.	.
	.	.	.	.	.	.	.	.	.

Medication (Name)	Dose (eg '5mg')	Frequency (eg 2 x / day)	Date Started	Date Stopped

If associated with TED or its treatment please record the cost (or prescription charge) of each purchase

£	.	.	.	.	.	.	.	.	.
	.	.	.	.	.	.	.	.	.

Medication (Name)	Dose (eg '5mg')	Frequency (eg 2 x / day)	Date Started	Date Stopped

If associated with TED or its treatment please record the cost (or prescription charge) of each purchase

£									
	.	.	.	.	.	.	.	.	.
	.	.	.	.	.	.	.	.	.

Medication (Name)	Dose (eg '5mg')	Frequency (eg 2 x / day)	Date Started	Date Stopped

If associated with TED or its treatment please record the cost (or prescription charge) of each purchase

£									
	.	.	.	.	.	.	.	.	.
	.	.	.	.	.	.	.	.	.

Medication (Name)	Dose (eg ‘5mg’)	Frequency (eg 2 x / day)	Date Started	Date Stopped

If associated with TED or its treatment please record the cost (or prescription charge) of each purchase

£	.	.	.	.	.	.	.	.	.
	.	.	.	.	.	.	.	.	.

Medication (Name)	Dose (eg ‘5mg’)	Frequency (eg 2 x / day)	Date Started	Date Stopped

If associated with TED or its treatment please record the cost (or prescription charge) of each purchase

£	.	.	.	.	.	.	.	.	.
	.	.	.	.	.	.	.	.	.

Medication (Name)	Dose (eg ‘5mg’)	Frequency (eg 2 x / day)	Date Started	Date Stopped

If associated with TED or its treatment please record the cost (or prescription charge) of each purchase

£	.	.	.	.	.	.	.	.	.
	.	.	.	.	.	.	.	.	.

Medication (Name)	Dose (eg ‘5mg’)	Frequency (eg 2 x / day)	Date Started	Date Stopped

If associated with TED or its treatment please record the cost (or prescription charge) of each purchase

£	.	.	.	.	.	.	.	.	.
	.	.	.	.	.	.	.	.	.

Health Record

It is important that we monitor all new health problems you develop during your participation in the trial. We are interested in any new symptom or illness, however minor; regardless of whether you feel this is related to your Thyroid Eye Disease and its treatment. For example, you should record if you develop heart-burn or tiredness, have a cold, or even are involved in an accident. The main reason for this is identify any potential side-effects of the medications you are taking. Please use the following table to keep an accurate account of this information.

New Symptom or Illness	Date Started (d / m / y)	Date Stopped (d / m / y)	Maximum Intensity (Mild / Moderate / Severe)

New Symptom or Illness	Date Started (d / m / y)	Date Stopped (d / m / y)	Maximum Intensity (Mild / Moderate / Severe)

New Symptom or Illness	Date Started (d / m / y)	Date Stopped (d / m / y)	Maximum Intensity (Mild / Moderate / Severe)

New Symptom or Illness	Date Started (d / m / y)	Date Stopped (d / m / y)	Maximum Intensity (Mild / Moderate / Severe)

New Symptom or Illness	Date Started (d / m / y)	Date Stopped (d / m / y)	Maximum Intensity (Mild / Moderate / Severe)

NHS / Trial Appointments

This section is a useful place to record the times and dates of your visits to hospital, GP or other healthcare services.

We would also like to use it to calculate:

- How much your journeys to attend your appointments cost,
- How much of your time this takes,
- Whether or not you incur additional costs (such as paying for the care of children or elderly relatives).

You may be accompanied by a friend or relative, and we would like to include their costs too.

There are separate sections for visits to the Eye Hospital, Radiotherapy, your Endocrinologist, General Practitioner and other NHS health care services (such as A&E).

Eye Hospital Appointments

Please record the following for each Eye Hospital appointment you attend

- **Travel Costs** = Total cost of *return* fares (eg taxi, train, bus, tube) and parking tickets *for you and all those accompanying you* (eg if you travel by train with your spouse to attend your appointment and the cost of your return train fare is £35 each, the total cost for the visit would be £70)
- **Miles** = If you completed all or part of your journey by car (or motorbike) please estimate the round trip distance in miles (leave blank if you did not drive)
- **No. of other People** = Number of people accompanying you (leave blank if you attended on your own)
- **Time Taken** = Total number of hours taken for your round trip (eg if you left home at 09:00 and get back at 15:00, the time taken for the visit would be 6 hours)
- **Days off Work** = Total time taken off work to attend the appointment *by you and those accompanying you* (eg if you take ¼ day and your spouse takes ½ day, the total = ¾ day),
- **Income Lost** = *If you, or any of the people accompanying you*, lost pay while attending your hospital appointment please estimate the total amount this cost (eg if you lost £100 and your spouse lost £50, the total = £150)
- **Hosp Trans** = Tick this box (and leave the travel costs / mileage sections blank) if you are disabled and use the hospital's transport services
- **Care Costs** = Complete this section if you pay for the care of dependent relatives (eg children or elderly parents) while you attend hospital appointments.
- **Overnight** = If you travel a long distance to each hospital visit and stay overnight to break up the journey, or to enable you to make a morning appointment, please record the cost of accommodation *for you and all those accompanying you*

EYE HOSPITAL

Date	Time	Travel Costs (£)	Miles	No. of Other People	Time Taken (hrs)	Days off Work	Income Lost (£)	Hosp Trans	Care Costs (£)	Over-night (£)
/ /								❒		
/ /								❒		
/ /								❒		
/ /								❒		
/ /								❒		
/ /								❒		
/ /								❒		
/ /								❒		

EYE HOSPITAL

Date	Time	Travel Costs (£)	Miles	No. of Other People	Time Taken (hrs)	Days off Work	Income Lost (£)	Hosp Trans	Care Costs (£)	Over-night (£)
/ /								☐		
/ /								☐		
/ /								☐		
/ /								☐		
/ /								☐		
/ /								☐		
/ /								☐		
/ /								☐		

Radiotherapy Appointments

Please record the following for each Radiotherapy appointment you attend

- **Travel Costs** = Total cost of *return* fares (eg taxi, train, bus, tube) and parking tickets *for you and all those accompanying you* (eg if you travel by train with your spouse to attend your appointment and the cost of your return train fare is £35 each, the total cost for the visit would be £70)
- **Miles** = If you completed all or part of your journey by car (or motorbike) please estimate the round trip distance in miles (leave blank if you did not drive)
- **No. of other People** = Number of people accompanying you (leave blank if you attended on your own)
- **Time Taken** = Total number of hours taken for your round trip (eg if you left home at 09:00 and get back at 15:00, the time taken for the visit would be 6 hours)
- **Days off Work** = Total time taken off work to attend the appointment *by you and those accompanying you* (eg if you take ¼ day and your spouse takes ½ day, the total = ¾ day),
- **Income Lost** = *If you, or any of the people accompanying you*, lost pay while attending your hospital appointment please estimate the total amount this cost (eg if you lost £100 and your spouse lost £50, the total = £150)
- **Hosp Trans** = Tick this box (and leave the travel costs / mileage sections blank) if you are disabled and use the hospital's transport services
- **Care Costs** = Complete this section if you pay for the care of dependent relatives (eg children or elderly parents) while you attend hospital appointments.
- **Overnight** = If you travel a long distance to each hospital visit and stay overnight to break up the journey, or to enable you to make a morning appointment, please record the cost of accommodation *for you and all those accompanying you*

RADIOTHERAPY

Date	Time	Travel Costs (£)	Miles	No. of Other People	Time Taken (hrs)	Days off Work	Income Lost (£)	Hosp Trans	Care Costs (£)	Over-night (£)
/ /								❒		
/ /								❒		
/ /								❒		
/ /								❒		
/ /								❒		
/ /								❒		
/ /								❒		
/ /								❒		

RADIOTHERAPY

Date	Time	Travel Costs (£)	Miles	No. of Other People	Time Taken (hrs)	Days off Work	Income Lost (£)	Hosp Trans	Care Costs (£)	Over-night (£)
/ /								❐		
/ /								❐		
/ /								❐		
/ /								❐		
/ /								❐		
/ /								❐		
/ /								❐		
/ /								❐		

Endocrinology Appointments

If you are seen by your local Endocrinologist in addition to Eye Hospital appointments, please record the details of these visits below:

- **Reason** = Tick the box if the appointment was to review the care your Thyroid Gland or Thyroid Eye Disease. Otherwise please write a brief description of the reason for the appointment or symptoms which caused you to seek the endocrinologist's advice in the 'other' box.
- **Travel Costs** = Total cost of *return* fares (eg taxi, train, bus, tube) and parking tickets *for you and all those accompanying you* (eg if you travel by train with your spouse to attend your appointment and the cost of your return train fare is £35 each, the total cost for the visit would be £70)
- **Miles** = If you completed all or part of your journey by car (or motorbike) please estimate the round trip distance in miles (leave blank if you did not drive)
- **No. of other People** = Number of people accompanying you (leave blank if you attended on your own)
- **Time Taken** = Total number of hours taken for your round trip (eg if you left home at 09:00 and get back at 15:00, the time taken for the visit would be 6 hours)
- **Days off Work** = Total time taken off work to attend the appointment *by you and those accompanying you* (eg if you take ¼ day and your spouse takes ½ day, the total = ¾ day),
- **Income Lost** = *If you, or any of the people accompanying you*, lost pay while attending your hospital appointment please estimate the total amount this cost (eg if you lost £100 and your spouse lost £50, the total = £150)
- **Hosp Trans** = Tick this box (and leave the travel costs / mileage sections blank) if you are disabled and use the hospital's transport services

ENDOCRINOLOGIST

Date	Time	Reason	Travel Costs (£)	Miles	No. of Other People	Time Taken (hrs)	Days off Work	Income Lost (£)	Hosp Trans
/ /		Thyroid ❐							❐
		Other:							
/ /		Thyroid ❐							❐
		Other:							
/ /		Thyroid ❐							❐
		Other:							

ENDOCRINOLOGIST

Date	Time	Reason	Travel Costs (£)	Miles	No. of Other People	Time Taken (hrs)	Days off Work	Income Lost (£)	Hosp Trans
/ /		Thyroid ❒							❒
		Other:							
/ /		Thyroid ❒							❒
		Other:							
/ /		Thyroid ❒							❒
		Other:							

General Practitioner (GP) Appointments

If you are seen by your GP in addition to Eye Hospital appointments, please record the details of these vistis below:

- **Reason**
  - Tick the ‘Thyroid’ box if the appointment was with your GP to review the care your Thyroid Gland or Thyroid Eye Disease
  - Tick the ‘Blood Test’ box if the appointment was with the *Practice Nurse* for a blood test to monitor your thyroid gland function or the effects of medications for your thyroid eye disease (including trial drugs) *and you did not see the GP*
  - Otherwise please write a brief description of the reason for the appointment or symptoms which caused you to seek your GP’s advice in the ‘other’ box.
- **Travel Costs** = Total cost of *return* fares (eg taxi, train, bus, tube) and parking tickets *for you and all those accompanying you* (eg if you travel by train with your spouse to attend your appointment and the cost of your return train fare is £35 each, the total cost for the visit would be £70)
- **Miles** = If you completed all or part of your journey by car (or motorbike) please estimate the round trip distance in miles (leave blank if you did not drive)
- **No. of other People** = Number of people accompanying you (leave blank if you attended on your own)
- **Time Taken** = Total number of hours taken for your round trip (eg if you left home at 09:00 and get back at 15:00, the time taken for the visit would be 6 hours)
- **Days off Work** = Total time taken off work to attend the appointment *by you and those accompanying you* (eg if you take ¼ day and your spouse takes ½ day, the total = ¾ day),
- **Income Lost** = *If you, or any of the people accompanying you*, lost pay while attending your hospital appointment please estimate the total amount this cost (eg if you lost £100 and your spouse lost £50, the total = £150)

GENERAL PRACTITIONER

Date	Time	Reason	Travel Costs (£)	Miles	No. of Other People	Time Taken (hrs)	Days off Work	Income Lost (£)
/ /		Thyroid ❐<br>Blood Tests ❐						
		Other:						
/ /		Thyroid ❐<br>Blood Tests ❐						
		Other:						
/ /		Thyroid ❐<br>Blood Tests ❐						
		Other:						

GENERAL PRACTITIONER

Date	Time	Reason	Travel Costs (£)	Miles	No. of Other People	Time Taken (hrs)	Days off Work	Income Lost (£)
/ /		Thyroid ❐<br>Blood Tests ❐						
		Other:						
/ /		Thyroid ❐<br>Blood Tests ❐						
		Other:						
/ /		Thyroid ❐<br>Blood Tests ❐						
		Other:						

GENERAL PRACTITIONER

Date	Time	Reason	Travel Costs (£)	Miles	No. of Other People	Time Taken (hrs)	Days off Work	Income Lost (£)
/ /		Thyroid ❐<br>Blood Tests ❐						
		Other:						
/ /		Thyroid ❐<br>Blood Tests ❐						
		Other:						
/ /		Thyroid ❐<br>Blood Tests ❐						
		Other:						

GENERAL PRACTITIONER

Date	Time	Reason	Travel Costs (£)	Miles	No. of Other People	Time Taken (hrs)	Days off Work	Income Lost (£)
/ /		Thyroid ☐<br>Blood Tests ☐						
[redacted]		Other:						
/ /		Thyroid ☐<br>Blood Tests ☐						
[redacted]		Other:						
/ /		Thyroid ☐<br>Blood Tests ☐						
[redacted]		Other:						

GENERAL PRACTITIONER

Date	Time	Reason	Travel Costs (£)	Miles	No. of Other People	Time Taken (hrs)	Days off Work	Income Lost (£)
/ /		Thyroid ❒<br>Blood Tests ❒						
		Other:						
/ /		Thyroid ❒<br>Blood Tests ❒						
		Other:						
/ /		Thyroid ❒<br>Blood Tests ❒						
		Other:						

GENERAL PRACTITIONER

Date	Time	Reason	Travel Costs (£)	Miles	No. of Other People	Time Taken (hrs)	Days off Work	Income Lost (£)
/ /		Thyroid ☐<br>Blood Tests ☐						
		Other:						
/ /		Thyroid ☐<br>Blood Tests ☐						
		Other:						
/ /		Thyroid ☐<br>Blood Tests ☐						
		Other:						

GENERAL PRACTITIONER

Date	Time	Reason	Travel Costs (£)	Miles	No. of Other People	Time Taken (hrs)	Days off Work	Income Lost (£)
/ /		Thyroid ❐<br>Blood Tests ❐						
		Other:						
/ /		Thyroid ❐<br>Blood Tests ❐						
		Other:						
/ /		Thyroid ❐<br>Blood Tests ❐						
		Other:						

GENERAL PRACTITIONER

Date	Time	Reason	Travel Costs (£)	Miles	No. of Other People	Time Taken (hrs)	Days off Work	Income Lost (£)
/ /		Thyroid ❐ Blood Tests ❐						
		Other:						
/ /		Thyroid ❐ Blood Tests ❐						
		Other:						
/ /		Thyroid ❐ Blood Tests ❐						
		Other:						

Hospital Admissions

If you have been admitted to hospital for any reason since entering the CIRTED trial, please record the details below

- **Reason**
    - Tick the 'Thyroid' box if the reason for your admission was related to your Thyroid Gland or Thyroid Eye Disease
    - Otherwise tick the 'Unrelated' box

  Regardless of which box you tick please also explain the reasons for your admission in more detail

- **Inpatient Days** = Number of days in hospital
- **Days off Work** = Time taken off work *by you* because of your hospital admission.
- **Income Lost** = Your Loss of Earnings as a result of your time off work
- **Care Costs** = Cost of paying for the care of dependent relatives (eg children or elderly parents) while you were in hospital
- **Cost to Others** = An estimate of the total cost to your friends and family as a result of your hospital stay (eg their travel costs and loss of earnings through time off work to visit you or care for your dependents)

Date of Admission	Reason	Inpatient Days	Days off Work	Income Lost (£)	Care Costs	Cost to Others
/ /	Thyroid ☐<br>Unrelated ☐<br>Details:					
/ /	Thyroid ☐<br>Unrelated ☐<br>Details:					

Date of Admission	Reason	Inpatient Days	Days off Work	Income Lost (£)	Care Costs	Cost to Others
/ /	Thyroid ❐<br>Unrelated ❐<br>Details:					
/ /	Thyroid ❐<br>Unrelated ❐<br>Details:					

Other NHS Contacts (including Phone Calls)

If you use other NHS services during your time in the CIRTED trial (such as Accident & Emergency, or phone calls to NHS Direct, any hospitals you attend), please record these below

- **Service** = Please tick the appropriate box
- **Reason**
    - Tick the 'Thyroid' box if the reason for using the service was related to your Thyroid Gland or Thyroid Eye Disease
    - Otherwise tick the 'Unrelated' box

  Regardless of which box you tick please also give a more detailed explanation

- **Time Taken** = Please estimate the total number of hours taken to use the service (including travelling time). For phone calls, estimate the length of the call (in minutes)
- **Travel Costs** = If you travelled to use the service what was total cost of *return* fares (eg taxi, train, bus, tube) and parking tickets *for you and all those accompanying you* (eg if you travel by train with your spouse to attend your appointment and the cost of your return train fare is £35 each, the total cost for the visit would be £70)
- **Miles** = If you travelled to use the service and completed all or part of your journey by car (or motorbike) please estimate the round trip distance in miles (leave blank if you did not drive)
- **Other People** = Number of people accompanying you to use the service (leave blank if you attended on your own)
- **Days off Work** = Total time taken off work to use this service *by you and those accompanying you* (eg if you take ¼ day and your spouse takes ½ day, the total = ¾ day)
- **Income Lost** = *If you, or any of the people accompanying you*, lost pay while using this NHS service please estimate the total amount this cost (eg if you lost £100 and your spouse lost £50, the total = £150)

Date	Service	Reason	Time Taken	*Travel Costs (£)	*Miles	*No. of Other People	*Days off Work	*Income Lost (£)
/ /	A&E ❐<br>Walk-in Centre ❐<br>NHS Direct ❐<br>Other PhoneCall ❐ (give details)<br>Other ❐ (give details)	Thyroid ❐<br>Unrelated ❐						
	Details:	Details:						

* Leave blank if phone-call

Date	Service	Reason	Time Taken	*Travel Costs (£)	*Miles	*No. of Other People	*Days off Work	*Income Lost (£)
/ /	A&E ❐<br>Walk-in Centre ❐<br>NHS Direct ❐<br>Other PhoneCall ❐<br>(give details)<br>Other ❐<br>(give details)	Thyroid ❐<br>Unrelated ❐						
	Details:	Details:						

* **Leave blank if phone-call**

Date	Service	Reason	Time Taken	*Travel Costs (£)	*Miles	*No. of Other People	*Days off Work	*Income Lost (£)
/ /	A&E ❒<br>Walk-in Centre ❒<br>NHS Direct ❒<br>Other PhoneCall ❒<br>(give details)<br>Other ❒<br>(give details)	Thyroid ❒<br>Unrelated ❒						
	**Details:**	**Details:**						

* **Leave blank if phone-call**

Date	Service	Reason	Time Taken	*Travel Costs (£)	*Miles	*No. of Other People	*Days off Work	*Income Lost (£)
/ /	A&E ❐<br>Walk-in Centre ❐<br>NHS Direct ❐<br>Other PhoneCall ❐<br>(give details)<br>Other ❐<br>(give details)	Thyroid ❐<br>Unrelated ❐						
	Details:	Details:						

* Leave blank if phone-call

Date	Service	Reason	Time Taken	*Travel Costs (£)	*Miles	*No. of Other People	*Days off Work	*Income Lost (£)
/ /	A&E ❐<br>Walk-in Centre ❐<br>NHS Direct ❐<br>Other PhoneCall ❐<br>(give details)<br>Other ❐<br>(give details)<br>Details:	Thyroid ❐<br>Unrelated ❐<br><br>Details:						

* Leave blank if phone-call

Date	Service	Reason	Time Taken	*Travel Costs (£)	*Miles	*No. of Other People	*Days off Work	*Income Lost (£)
/ /	A&E ☐<br>Walk-in Centre ☐<br>NHS Direct ☐<br>Other PhoneCall ☐ (give details)<br>Other ☐ (give details)	Thyroid ☐<br>Unrelated ☐						
	Details:	Details:						

* **Leave blank if phone-call**

Use of Non- NHS Services

If you use other Non-NHS health services during your time in the CIRTED trial (such as private medical consultations, complimentary therapy or a patient support group) please record these below

- **Service** = Please tick the appropriate box and give a more detailed description of the service
- **Reason**
  - Tick the 'Thyroid' box if the reason for using the service was related to your Thyroid Gland or Thyroid Eye Disease
  - Otherwise tick the 'Unrelated' box

  Regardless of which box you tick please also give a more detailed explanation

- **Cost** = Cost of the service for that visit
- **Time Taken** = Please estimate the total number of hours taken to use the service (including travelling time).
- **Travel** = If you travelled to use the service what was total cost of *return* fares (eg taxi, train, bus, tube) and parking tickets *for you and all those accompanying you* (eg if you travel by train with your spouse to attend your appointment and the cost of your return train fare is £35 each, the total cost for the visit would be £70)
- **Miles** = If you travelled to use the service and completed all or part of your journey by car (or motorbike) please estimate the round trip distance in miles (leave blank if you did not drive)
- **No. of Other People** = Number of people accompanying you to use the service (leave blank if you attended on your own)
- **Days off Work** = Total time taken off work to use this service *by you and those accompanying you* (eg if you take ¼ day and your spouse takes ½ day, the total = ¾ day)
- **Income Lost** = *If you, or any of the people accompanying you*, lost pay while using this NHS service please estimate the total amount this cost (eg if you lost £100 and your spouse lost £50, the total = £150)

Date	Service	Reason	Cost (£)	Time Taken (hrs)	Travel (£)	Miles	No. of Other People	Days off Work	Income Lost (£)
/ /	Private Medical ❐<br>Acupuncture ❐<br>Counsellor ❐<br>Support Group ❐<br>Other ❐	Thyroid ❐<br>Unrelated ❐							
	Details:	Details:							

Date	Service	Reason	Cost (£)	Time Taken (hrs)	Travel (£)	Miles	No. of Other People	Days off Work	Income Lost (£)
/ /	Private Medical ☐<br>Acupuncture ☐<br>Counsellor ☐<br>Support Group ☐<br>Other ☐	Thyroid ☐<br>Unrelated ☐							
	Details:	Details:							

Date	Service	Reason	Cost (£)	Time Taken (hrs)	Travel (£)	Miles	No. of Other People	Days off Work	Income Lost (£)
/ /	Private Medical ☐<br>Acupuncture ☐<br>Counsellor ☐<br>Support Group ☐<br>Other ☐	Thyroid ☐<br>Unrelated ☐							
	Details:	Details:							

Date	Service	Reason	Cost (£)	Time Taken (hrs)	Travel (£)	Miles	No. of Other People	Days off Work	Income Lost (£)
/ /	Private Medical ☐<br>Acupuncture ☐<br>Counsellor ☐<br>Support Group ☐<br>Other ☐	Thyroid ☐<br>Unrelated ☐							
	Details:	Details:							

Date	Service	Reason	Cost (£)	Time Taken (hrs)	Travel (£)	Miles	No. of Other People	Days off Work	Income Lost (£)
/ /	Private Medical ☐<br>Acupuncture ☐<br>Counsellor ☐<br>Support Group ☐<br>Other ☐	Thyroid ☐<br>Unrelated ☐							
	Details:	Details:							

Date	Service	Reason	Cost (£)	Time Taken (hrs)	Travel (£)	Miles	No. of Other People	Days off Work	Income Lost (£)
/ /	Private Medical ☐<br>Acupuncture ☐<br>Counsellor ☐<br>Support Group ☐<br>Other ☐	Thyroid ☐<br>Unrelated ☐							
	Details:	Details:							

Date	Service	Reason	Cost (£)	Time Taken (hrs)	Travel (£)	Miles	No. of Other People	Days off Work	Income Lost (£)
/ /	Private Medical ☐<br>Acupuncture ☐<br>Counsellor ☐<br>Support Group ☐<br>Other ☐	Thyroid ☐<br>Unrelated ☐							
	Details:	Details:							

Date	Service	Reason	Cost (£)	Time Taken (hrs)	Travel (£)	Miles	No. of Other People	Days off Work	Income Lost (£)
/ /	Private Medical ❒<br>Acupuncture ❒<br>Counsellor ❒<br>Support Group ❒<br>Other ❒<br><br>Details:	Thyroid ❒<br>Unrelated ❒<br><br>Details:							

Date	Service	Reason	Cost (£)	Time Taken (hrs)	Travel (£)	Miles	No. of Other People	Days off Work	Income Lost (£)
/ /	Private Medical ☐<br>Acupuncture ☐<br>Counsellor ☐<br>Support Group ☐<br>Other ☐	Thyroid ☐<br>Unrelated ☐							
	Details:	Details:							

Date	Service	Reason	Cost (£)	Time Taken (hrs)	Travel (£)	Miles	No. of Other People	Days off Work	Income Lost (£)
/ /	Private Medical ☐<br>Acupuncture ☐<br>Counsellor ☐<br>Support Group ☐<br>Other ☐	Thyroid ☐<br>Unrelated ☐							
	Details:	Details:							

Date	Service	Reason	Cost (£)	Time Taken (hrs)	Travel (£)	Miles	No. of Other People	Days off Work	Income Lost (£)
/ /	Private Medical ☐<br>Acupuncture ☐<br>Counsellor ☐<br>Support Group ☐<br>Other ☐	Thyroid ☐<br>Unrelated ☐							
	Details:	Details:							

Thyroid Eye Disease (TED) Related Expenses

Please record any purchases, or other expenses, related to your TED (eg new glasses incorporating prisms, sunglasses, or cosmetics)

Date	Item	Reason related to TED	Cost (£)
/ /	Glasses ❒ Cosmetics ❒ Other ❒		
[redacted]	Details:	[redacted]	[redacted]

Date	Item	Reason related to TED	Cost (£)
/ /	Glasses ❐ Cosmetics ❐ Other ❐<br>Details:		

Date	Item	Reason related to TED	Cost (£)
/ /	Glasses ❐ Cosmetics ❐ Other ❐<br>Details:		

Date	Item	Reason related to TED	Cost (£)
/ /	Glasses ❐ Cosmetics ❐ Other ❐<br>Details:		

Date	Item	Reason related to TED	Cost (£)
/ /	Glasses ❐ Cosmetics ❐ Other ❐<br>Details:		

Date	Item	Reason related to TED	Cost (£)
/ /	**Glasses** ☐ **Cosmetics** ☐ **Other** ☐<br>**Details:**		

Date	Item	Reason related to TED	Cost (£)
/ /	**Glasses** ☐ **Cosmetics** ☐ **Other** ☐<br>**Details:**		

Date	Item	Reason related to TED	Cost (£)
/ /	Glasses ❐ Cosmetics ❐ Other ❐<br>Details:		

Date	Item	Reason related to TED	Cost (£)
/ /	Glasses ❐ Cosmetics ❐ Other ❐<br>Details:		

Date	Item	Reason related to TED	Cost (£)
/ /	Glasses ❒ Cosmetics ❒ Other ❒<br>Details:		

Date	Item	Reason related to TED	Cost (£)
/ /	Glasses ❒ Cosmetics ❒ Other ❒<br>Details:		

Date	Item	Reason related to TED	Cost (£)
/ /	Glasses ❐ Cosmetics ❐ Other ❐<br>Details:		

Date	Item	Reason related to TED	Cost (£)
/ /	Glasses ❐ Cosmetics ❐ Other ❐<br>Details:		

Date	Item	Reason related to TED	Cost (£)
/ /	Glasses ❐ Cosmetics ❐ Other ❐<br><br>Details:		

Date	Item	Reason related to TED	Cost (£)
/ /	Glasses ❐ Cosmetics ❐ Other ❐<br><br>Details:		

Date	Item	Reason related to TED	Cost (£)
/ /	Glasses ❐ Cosmetics ❐ Other ❐<br>Details:		

Date	Item	Reason related to TED	Cost (£)
/ /	Glasses ❐ Cosmetics ❐ Other ❐<br>Details:		

Notes

Please record any additional notes, either for your own benefit or comments which you think will be of interest to the CIRTED trial investigators, on the following pages.

NATIONAL EYE RESEARCH CENTRE

Registered Charity No. 294087

University of BRISTOL
