# Supplementary material for: Protocol for the combined immunosuppression & radiotherapy in thyroid eye disease (CIRTED) trial: A multi-centre, double-masked, factorial randomised controlled trial
Source: Trials. 2008 Jan 31;9:6. doi: 10.1186/1745-6215-9-6 (PMC2275219; doi:10.1186/1745-6215-9-6)
Supplement: Additional file 2 — Quality of Life Questionnaires. Questionnaire to assess the patent's quality of life. This material uses the WHOQOL-UK and the assistance of the University of Bath and the World Health Organisation is acknowledged. [file 1745-6215-9-6-S2.pdf]

Combined Immunosuppression and Radiotherapy in Thyroid Eye Disease (CIRTED)

Health Related Quality of Life

Dear Participant,

Thank you for agreeing to complete the following questionnaire as part of the CIRTED trial; your views are very valuable to us.

These questionnaires are designed to examine your personal views regarding your quality of life, health and other areas. You can be assured that your responses to the questions within this pack will remain confidential and your anonymity safeguarded.

Please check that you have answered all the questions before returning the questionnaires to the Researcher.

Many thanks for your cooperation.

Researchers at the Centre for Appearance Research
University of the West of England, Bristol

Professor Nichola Rumsey
(Nichola.Rumsey@uwe.ac.uk)
Director - Centre for Appearance Research

Sue Jackson
(sue@winterbourne.freeserve.co.uk)
Research Fellow

Laura Kingston
(Laura2.Kingston@uwe.ac.uk)
Research Associate

UWE BRISTOL University of the West of England

In association with Bristol Eye Hospital and Moorfields Eye Hospital, London

Date____________ Code ____________

Section A: Quality of Life

Please answer all the questions. If you are unsure about which response to give to a question, please choose the one that appears most appropriate. This can often be your first response. Please keep in mind your standards, hopes, pleasures and concerns.

We ask that you think about your life in the last two weeks and circle the number on the scale for each question that gives the best answer for you as it relates to your thyroid eye disease.

A1.	Very Poor	Poor	Neither poor nor good	Good	Very good
How would you rate your quality of life?	1	2	3	4	5

A2.	Very dissatisfied	Dissatisfied	Neither satisfied nor dissatisfied	Satisfied	Very satisfied
How satisfied are you with your health?	1	2	3	4	5

The following questions ask about how much you have experienced things in the last two weeks.

A3.	Not at all	A little	A moderate amount	Very much	An extreme amount
How much do you feel that physical pain prevents you from doing what you need to do?	1	2	3	4	5
A4.					
How much do you need any medical treatment to function in your daily life?	1	2	3	4	5
A5.					
How much do you enjoy life?	1	2	3	4	5
A6.					
To what extent do you feel life to be meaningful?	1	2	3	4	5

A7.	Not at all	A little	A moderate amount	Very much	Extremely
How well are you able to concentrate?	1	2	3	4	5
A8.					
How safe do you feel in your daily life?	1	2	3	4	5
A9.					
How healthy is your physical environment (eg housing)?	1	2	3	4	5

Date_____________ Code _____________

The following questions ask about how completely you experienced or were able to do certain things in the last two weeks.

	Not at all	A little	Moderately	Mostly	Completely
A10.					
Do you have enough energy for everyday life?	1	2	3	4	5
A11.					
Are you able to accept your bodily appearance?	1	2	3	4	5
A12.					
To what extent do you have enough money to meet your needs?	1	2	3	4	5
A13.					
How available to you is the information that you need in your day-to-day life?	1	2	3	4	5
A14.					
To what extent do you have the opportunity for leisure activities?	1	2	3	4	5

A15.	Very Poor	Poor	Neither poor nor good	Good	Very good
How well are you able to get around?	1	2	3	4	5

The following questions ask you to say how good or satisfied you have felt about various aspects of your life over the last two weeks.

A16.	Very dissatisfied	Dissatisfied	Neither satisfied nor dissatisfied	Satisfied	Very satisfied
How satisfied are you with your sleep?	1	2	3	4	5
A17.					
How satisfied are you with your ability to perform daily living activities?	1	2	3	4	5
A18.					
How satisfied are you with your capacity for work?	1	2	3	4	5
A19.					
How satisfied are you with yourself?	1	2	3	4	5
A20.					
How satisfied are you with your personal relationships?	1	2	3	4	5
A21.					
How satisfied are you with your sex life?	1	2	3	4	5
A22.					
How satisfied are you with the support you get from your friends?	1	2	3	4	5

Date____________ Code ____________

The following questions ask you to say how good or satisfied you have felt about various aspects of your life over the last two weeks.

A23.	Very dissatisfied	Dissatisfied	Neither satisfied nor dissatisfied	Satisfied	Very satisfied
How satisfied are you with the conditions of your living place?	1	2	3	4	5
A24.					
How satisfied are you with your access to health services?	1	2	3	4	5
A25.					
How satisfied are you with your transport?	1	2	3	4	5

The following questions refer to how often you have felt or experienced certain things in the last two weeks.

A26.	Never	Seldom	Quite Often	Very Often	Always
How often do you have negative feelings, such as blue mood, despair, anxiety, depression?	1	2	3	4	5

A27.	Not at all	Not much	A moderate amount	Very much	An extreme amount
How fed up do you feel?	1	2	3	4	5

A28.	Very dissatisfied	Dissatisfied	Neither satisfied nor dissatisfied	Satisfied	Very Satisfied
How satisfied are you with your level of happiness?	1	2	3	4	5

The following two questions with a long black line require you to put a slash through the line with a pen. For example a slash marked at this point on the line would mean that you feel your thyroid eye disease is “quite noticeable” to other people but not to such an extent as “very noticeable”.

Not at all noticeable ———————————/——————— ***Very noticeable***

N. How noticeable do you feel your thyroid eye disease is to other people?

Not at all noticeable ———————————————— Very noticeable

S. How severe do you feel your thyroid eye disease is?

Not very severe ———————————————— Extremely severe

Date____________ Code ____________

Section B: Appearance

The following questions are concerned with the way you feel or act. They are all simple. Please tick the answer that applies to you. If the item does not apply to you at all, please tick the N/A (Not Applicable) option. Do not spend long on any one question.

Please remember: These questions should be answered in relation to how your thyroid eye disease may or may not affect you.

B1. How confident do you feel?

Not at all ☐ Slightly ☐ Moderately ☐ Extremely ☐

B2. How distressed do you get when you see yourself in the mirror/window?

Extremely ☐ Moderately ☐ A little ☐ Not at all distressed ☐

B3. My self-consciousness makes me irritable at home:

N/A ☐ Never/Almost Never ☐ Sometimes ☐ Often ☐ Almost Always ☐

B4. How hurt do you feel?

Extremely ☐ Moderately ☐ Slightly ☐ Not at all ☐

B5. At present my self-consciousness has an adverse effect on my work:

Almost Always ☐ Often ☐ Sometimes ☐ Never/Almost Never ☐ N/A ☐

B6. How distressed do you get when you go to the beach?

N/A ☐ Not at all ☐ A little ☐ Moderately ☐ Extremely ☐

B7. Other people misjudge me because of my eye condition:

Almost Always ☐ Often ☐ Sometimes ☐ Never/Almost Never ☐ N/A ☐

B8. How feminine/masculine do you feel?

Not at all ☐ Slightly ☐ Moderately ☐ Extremely ☐

B9. I am self-conscious of my eye condition:

N/A ☐ Never/Almost Never ☐ Sometimes ☐ Often ☐ Almost Always ☐

B10. How irritable do you feel?

Not at all ☐ Slightly ☐ Moderately ☐ Extremely ☐

B11. I adopt certain behaviours to conceal my eye condition (e.g. wearing dark glasses):

Never/Almost Never ☐ Sometimes ☐ Often ☐ Almost Always ☐

B12. I avoid communal changing rooms:

Almost Always ☐ Often ☐ Sometimes ☐ Never/Almost Never ☐ N/A ☐

B13. How distressed do you get by shopping in department stores/supermarkets?

N/A ☐ Not at all ☐ Slightly ☐ Moderately ☐ Extremely ☐

B14. How rejected do you feel?

Not at all ☐ Slightly ☐ Moderately ☐ Extremely ☐

B15. I avoid undressing in front of my partner:

N/A ☐ Never/Almost Never ☐ Sometimes ☐ Often ☐ Almost Always ☐

B16. How distressed do you get while playing sports/games?

Extremely ☐ Moderately ☐ Slightly ☐ Not at all ☐ N/A ☐

B17. I close into my shell:

Almost Always ☐ Often ☐ Sometimes ☐ Never/Almost Never ☐

B18. How distressed are you by feeling you want to conceal your eye condition?

Extremely ☐ Moderately ☐ Slightly ☐ Not at all ☐ N/A ☐

B19. How distressed do you get when going to social events?

N/A ☐ Not at all ☐ Moderately ☐ A fair amount ☐ Extremely ☐

B20. How normal do you feel?

Not at all ☐ Slightly ☐ Moderately ☐ Extremely ☐

B21. At present my self-consciousness has an adverse effect on my sex life:

Almost Always ☐ Often ☐ Sometimes ☐ Never/Almost Never ☐ N/A ☐

B22. I avoid going out of the house:

Almost Always ☐ Often ☐ Sometimes ☐ Never/Almost Never ☐

B23. How distressed do you get when other people make remarks about your eye condition**?**

N/A ☐ Not at all ☐ Moderately ☐ A fair amount ☐ Extremely ☐

B24. I avoid going out to pubs/restaurants/clubs:

Almost Always ☐ Often ☐ Sometimes ☐ Never/Almost Never ☐ N/A ☐

o My eye condition causes me physical pain/discomfort:

Never/Almost never ☐ Sometimes ☐ Often ☐ Almost always ☐

o My eye condition limits my <u>physical</u> ability to do the things I want to do:

Almost always ☐ Often ☐ Sometimes ☐ Never/Almost never ☐

Date____________ Code ____________

Section C: Thyroid Eye Disease

The following questions deal specifically with your thyroid eye disease.

Please focus on the past week while answering these questions.

During the past week, to what extent were you limited in carrying out the following activities, because of your thyroid eye disease?

Tick the box that matches your answer. The boxes correspond with the answers above them. Please tick only one box for each question.

	**Yes, severely limited**	**Yes, a little limited**	**No, not at all limited**		
C1. Bicycling	☐	☐	☐	Never learned to ride a bike	☐
C2. Driving	☐	☐	☐	No driver's licence	☐
C3. Moving around the house	☐	☐	☐		
C4. Walking outside	☐	☐	☐		
C5. Reading	☐	☐	☐		
C6. Watching TV	☐	☐	☐		
C7. Hobby or pastime, i.e.	☐	☐	☐		

	**Yes, severely hindered**	**Yes, a little hindered**	**No, not at all hindered**
C8. **During the past week**, did you feel hindered from doing something you wanted to do because of your thyroid eye disease?	☐	☐	☐

Date____________ Code ____________

The following questions deal with your thyroid eye disease <u>in general</u>

	Yes, very much so	Yes, a little	No, not at all
C9. Do you feel that your appearance has changed because of your thyroid eye disease?	☐	☐	☐
C10. Do you feel that you are stared at in the streets because of your thyroid eye disease?	☐	☐	☐
C11. Do you feel that people react unpleasantly because of your thyroid eye disease?	☐	☐	☐
C12. Do you feel that your thyroid eye disease has an influence on your self-confidence?	☐	☐	☐
C13. Do you feel that your thyroid eye disease has an influence on making friends?	☐	☐	☐
C14. Do you feel socially isolated because of your thyroid eye disease?	☐	☐	☐
C15. Do you feel that you appear less often in photos than before you had thyroid eye disease?	☐	☐	☐
C16. Do you try to mask changes in your appearance caused by thyroid eye disease?	☐	☐	☐

Date____________ Code ____________

Section D: How You Feel

Clinicians are well aware that emotions play an important part in most illnesses. Do not take too long over your replies; you immediate reaction to each item will probably be more accurate that a long thought-out response.

Read each item and underline the reply which comes closest to how you have been feeling in the last week.

D1. I feel tense or 'wound up':	Most of the time<br>A lot of the time<br>From time to time, occasionally<br>Not at all
D2. I still enjoy the things I used to enjoy:	Definitely as much<br>Not quite so much<br>Only a little<br>Hardly at all
D3. I get a sort of frightened feeling as if something awful is about to happen:	Very definitely and quite badly<br>Yes, but not too badly<br>A little, but it doesn't worry me<br>Not at all
D4. I can laugh and see the funny side of things:	As much as I always could<br>Not quite so much now<br>Definitely not so much now<br>Not at all
D5. Worrying thoughts go through my mind:	A great deal of the time<br>A lot of the time<br>From time to time but not too often<br>Only occasionally
D6. I feel cheerful:	Not at all<br>Not often<br>Sometimes<br>Most of the time

D7. I can sit at ease and feel relaxed:
- Definitely
- Usually
- Not often
- Not at all

D8. I feel as if I am slowed down:
- Nearly all the time
- Very often
- Sometimes
- Not at all

D9. I get a sort of frightened feeling like 'butterflies' in the stomach:
- Not at all
- Occasionally
- Quite often
- Very often

D10. I have lost interest in my appearance:
- Definitely
- I don't take as much care as I should
- I may not take quite as much care
- I take as much care as ever

D11. I feel restless as if I have to be on the move:
- Very much indeed
- Quite a lot
- Not very much
- Not at all

D12. I look forward with enjoyment to things:
- As much as ever I did
- Rather less than I used to
- Definitely less than I used to
- Hardly at all

D13. I get sudden feelings of panic:
- Very often indeed
- Quite often
- Not very often
- Not at all

D14. I can enjoy a good book, or radio, or TV programme:
- Often
- Sometimes
- Not often
- Very seldom

Date____________ Code ____________

E1. Does your thyroid eye disease impact your daily living in any way other than those described here?

____

____

____

E2. Did someone help you to fill out this questionnaire? **Yes** ☐ **No** ☐

E3. If '**Yes**', who assisted you? ____

E4. Do you have any comments about this questionnaire?

____

____

____

Many thanks for your invaluable participation

Please now return your fully completed questionnaire to the Researcher

If you are concerned by any issues raised by this questionnaire and you would like to discuss them further, please do not hesitate to contact us at the Centre for Appearance Research and we will be happy to put you in touch with the appropriate services.

The Centre for Appearance Research (CAR)
Faculty of Applied Sciences
University of the West of England (UWE, Bristol)
Frenchay Campus
Coldharbour Lane
Bristol BS16 1QY
t. 0117 328 3967
e. Laura2.Kingston@uwe.ac.uk
w. http://science.uwe.ac.uk/research/
