# Supplementary material for: Protocol for the combined immunosuppression & radiotherapy in thyroid eye disease (CIRTED) trial: A multi-centre, double-masked, factorial randomised controlled trial
Source: Trials. 2008 Jan 31;9:6. doi: 10.1186/1745-6215-9-6 (PMC2275219; doi:10.1186/1745-6215-9-6)
Supplement: Additional file 3 — Standard Operating Procedures. Standard operating procedures for the combined immunosuppression & radiotherapy in thyroid eye disease (CIRTED) trial. This material is a modification of the Standard Operating Procedures advocated by the European Group on Graves Ophthalmopathy (EUGOGO), and utilises Dickinson & Perros's reference atlas of colour plates [43]. [file 1745-6215-9-6-S3.pdf]

Standard Operating Procedures for the Combined Immunosuppression & Radiotherapy in Thyroid Eye Disease (CIRTED) Trial

Based on descriptions and photographic plates from:

- Dickinson AJ and Perros P. Controversies in the clinical evaluation of active thyroid associated orbitopathy: use of a detailed protocol with comparative photographs for objective assessment. *Clinical Endocrinology* 2001; 55: 283-303
- www.eugogo.org

Index

Standard Operating Procedure for Scoring Patients' Subjective Assessment of their Thyroid Eye Disease 2
Standard Operating Procedure for Examining Eyelids 3
Standard Operating Procedure for Assessing Conjunctival Redness and Lateral Rectus Injection 4
Standard Operating Procedure for Assessing Chemosis 5
Standard Operating Procedure for Assessing Caruncle Swelling 6
Standard Operating Procedure for Assessing the Cornea 7
Standard Operating Procedure for Applanation Tonometry 8
Standard Operating Procedure for Measuring Palpebral Aperture 10
Standard Operating Procedure for Exophthalmometry 11
Calculating Uniocular Fields of Fixation 12
Calculating Disease Activity (Clinical Activity Score) 14
Calculating Disease Severity (Total Eye Score) 15

Standard Operating Procedure for Scoring Patients' Subjective Assessment of their Thyroid Eye Disease

- *Pain / Oppressive Feeling:*

  Ask the patient if they have experienced any pain or pressure-like feeling behind their eye in the last 4 weeks

  If the patient reports any pain/oppressive feeling within the last 4 weeks, score 'YES'

  If no pain/oppressive feeling or unsure, score 'NO'

- *Pain on Eye Movements:*

  Ask the patient if they have experienced any pain when moving their eyes in the last 4 weeks

  If the patient reports any pain on eye movement in the last 4 weeks score 'YES'

  If no pain or unsure score 'NO'

- *Gorman Diplopia[*] Scoring*

  Ask the patient if they have experienced any double vision in the last 4 weeks

  If the patient reports never having diplopia, score as 'NONE'

  If the patient reports diplopia which is occasionally there (eg when fatigued) score as 'INTERMITTENT'

  If diplopia is always present but only gaze-evoked (not in primary position) score as 'INCONSTANT'

  If diplopia is permanently present (ie, in primary position, requires prisms or present in reading position) score as 'CONSTANT'

[*]Bahn R, Gorman,CA,. Choice of therapy and criteria for assessing treatment outcome in thyroid-associated ophthalmopathy. *Endocrinology and metabolism clinics of North America* 1987;16(2):391-407)

Standard Operating Procedure for Examining Eyelids

- Sit the patient on a chair in a well lit room
- Observe the patient from 1m away
- Look at the amount of eyelid swelling
- If the patient feels the swelling of their eyelids have not changed since having thyroid eye disease then score 'NO'. If you are unsure if the swelling is due to thyroid eye disease, score 'NO'

If there is:

a) only probable subcutaneous fluid or skin thickening, score 'NO' (see plates 2a[i], 2a[ii])

b) definite swelling or thickening with the skin tense or not tense, score 'YES' (see plates 2b[i], 2b[ii], 2c[i], 2c[ii]

- Compare colour of the eyelids to the rest of the patient's face
- Look for other causes of erythema and score 'NO' if predominantly due to another cause eg margin inflammation (see plate 1a)
- If there is redness due to their thyroid eye disease and the patient feels this has developed since having thyroid eye disease score 'YES' (see plate 1b[i], 1b[ii])

Standard Operating Procedure for Assessing Conjunctival Redness and Lateral Rectus Injection

- Sit the patient on a chair in a well lit room
- Ask the patient to fixate on a distant target straight ahead. With the patient looking in the primary position of gaze observe the area of visible conjunctiva in the palpebral aperture (if abnormal head posture cover one eye while observing opposite side)

If there is:

a) no redness, minimal redness or the redness of the conjunctiva is thought to be due to another cause such as exposure, score 'No'

b) definite redness which covers the equivalent of <50% of the visible conjunctiva, or the blood vessels are not dilated (not only involving the lateral areas), score 'YES' (see plate 3b[i] & 3b[ii])

c) redness covers the equivalent of <50% of the visible conjunctiva but the blood vessels are dilated, score 'YES' (see plate 3c[i])

d) <50% of the area of visible conjunctiva is red with significantly dilated blood vessels, score 'YES' (see plate 3d[i] 7 3d[ii])

- Ask the patient to look to their left and look at their right eye

  Separate the lateral eyelids

  If there are dilated vessels over the lateral rectus insertion score as 'YES'

  If there are no dilated vessels over the lateral rectus insertion score as 'NO'

  Ask the patient to look to their right and score the left eye

Standard Operating Procedure for Assessing Chemosis

- Sit the patient in front of a slit lamp. Ensure that the slit lamp is switched on and that the eye pieces are focused correctly.
- Ensure that the patient is not wearing a contact lens.
- The patient and slit lamp should be adjusted so that the patient's head is firmly positioned on the chin rest and against the forehead rest without leaning forward or straining.
- Instruct the patient to look straight ahead with both eyes wide open.
- Adjust the angle between the illumination and observation arms of the slit lamp to about 60 degrees and focus midway between the lateral canthus and the limbus with the eye in primary gaze.
- If there is:

a) No separation of the conjunctiva and sclera, score 'NO'

b) If the separation of the conjunctiva and and sclera in the vertical plane is less than 30% of the palpebral aperture and does not prolapse over the grey line, score 'NO' (see plate 5a)

c) If the separation is > 30% (or <30% and prolapsing over the grey line), score 'YES' (see plate 5b)

Standard Operating Procedure for Assessing Caruncle Swelling

- Sit the patient in front of a slit lamp
- Ensure that the slit lamp is switched on and that the eye pieces are focused correctly
- Adjust the angle between the illumination and observation arms of the slit lamp to about 60 degrees
- Ensure that the patient is not wearing a contact lens
- The patient and slit lamp should be adjusted so that the patient's head is firmly positioned on the chin rest and against the forehead rest without leaning forward or straining
- Instruct the patient to look straight ahead with both eyes wide open
- Look at the caruncle and plica of the right eye and then the left. If the caruncle or the plica are:

  a) not swollen or red or you are unsure, score 'NO'

  b) inflamed or red (see plate 4a & c), score 'YES'

  c) prolapse through closed eyelids, score 'YES' (see plate 4b)

Standard Operating Procedure for Assessing the Cornea

- Sit the patient in front of a slit lamp
- Ensure that the slit lamp is switched on and that the eye pieces are focused correctly
- Ensure that the patient is not wearing a contact lens
- The patient and slit lamp should be adjusted so that the patient's head is firmly positioned on the chin rest and against the forehead rest without leaning forward or straining
- Instruct the patient to look straight ahead with both eyes wide open
- Adjust the angle between the illumination and observation arms of the slit lamp to about 45 degrees
- Apply a fluoret or 2% fluorescein to both eyes
- With the blue filter on look at the right eye and then the left eye

If there is:

a) no staining, score 'absent'

b) evidence of punctuate/diffuse staining, score 'stippling'

c) an area of staining which is more than punctuate staining +/- corneal opacity, score 'ulcer'. The patient must leave the trial

d) any evidence of a perforation score it as such. The patient must leave the trial

Standard Operating Procedure for Applanation Tonometry

Goldmann applanation tonometry measures the intra-ocular pressure indirectly by measuring the force necessary to flatten a 3.06mm portion of the corneal surface. The higher the intra-ocular pressure, the greater the force required.

- Check that the Goldmann tonometer has been calibrated (this should be done weekly)
- Ensure that the slit lamp is switched on and that the eye pieces are focused correctly
- Insert the prism into the tonometer head and align the 0 on the prism with the white line on the tonometer head
- Switch the blue filter on and bring into the beam of the slit lamp
- Adjust the angle between the illumination and the slit lamp to about 60 degrees
- Insert the tonometer into base plate, it can be placed in two positions; one for each slit lamp eye-piece
- Ensure that the patient is not wearing a contact lens
- Instil one drop of Proxymetacaine 5% & Fluorescein 0.25% into both eyes
- The patient and slit lamp should be adjusted so that the patient's head is firmly positioned on the chin rest and against the forehead rest without leaning forward or straining. Tight fitting neckwear should be loosened.
- Both eyes will be tested, with the right eye preceding the left eye.
- Instruct the patient to look straight ahead with both eyes wide open, the patient's eyelids can be held open without placing any pressure on the eyeball.
- The examiner moves the tonometer forward and brings the prism into contact with the centre of the cornea, at this point the blue light illuminates the limbus.
- Upon contact with the cornea, a thin circular outline of fluorescein is produced. When viewed through the appropriate eye-piece of the slit lamp the prism splits the circle into two green coloured semicircles. It is important to ensure that these semicircles are the correct width as errors will occur if there is too much or too little fluorescein in the tear film. The control lever on the slit lamp is adjusted until the two semicircles are of equal size and in the middle of the field of vision.

- The tonometer’s dial is turned until the inner margins of each semi circle touch. This reading is then recorded as the intraocular pressure in primary gaze.
- Instruct the patient to look up as high as they can keeping their head straight. Repeat as above. This is recorded as the intraocular pressure in upgaze.

Standard Operating Procedure for Measuring Palpebral Aperture

- With the patient sitting on a chair positioned so the patient's eye level is at the same as the examiners.
- Ask the patient to fixate on the bridge of the examiner's nose. Try to correct an abnormal head posture or ocular alignment (if due to strabismus occlude the contralateral eye)
- Using a ruler place it vertically next to the midpupillary axis of the eye
- Look at the right eye first. Measure the palpebral aperture in millimetres.
- Repeat for the left eye

Standard Operating Procedure for Assessing Fixed Globe

- If the patient is unable to move the globe in any direction record as 'YES'
- If the globe can move and is freely mobile or only restricted in certain directions record 'NO'

Standard Operating Procedure for Exophthalmometry

Using the Oculus Hertel–type exophthalmometer

- Open it wider than required.

- Sit opposite the patient with the observer's and patient's eyes at the same level

- Keep the patient relaxed, avoiding breath holding and excessive eyelid retraction.

- Position left foot of Hertel against the patient's right lateral orbital rim, at level of lateral canthus. It should sit firmly as medially as possible, but outside lateral canthus and without distorting position of globe.

- Slide right foot medially into identical postion on left orbital rim. This will feel tight and slightly uncomfortable, but minimises potential side slippage of Hertel.

- Ask patient to fix their right eye on your left eye while you occlude the patient's left visual axis with your right thumb. In this position, align the instrument such that the vertical mark (or arrow) is aligned with the manufacturer's pre-marked position on the ruler. Once aligned, rotate the instrument slightly around the horizontal plane such as to view the apex of the cornea in the mirror and record the position of the corneal apex.
- To record the left eye, hold the instrument stationary and move your head. Then use your right eye to record the patients left eye. Again, the opposite visual axis is occluded by your left thumb, while the patient is asked to fix on your right eye. Ensure that the corneal apex is measured by rotating the instrument slightly around the horizontal plane if required.

Calculating Uniocular Fields of Fixation +

Measure the distance from the centre of the field sheet (from 0o) to the edge of the shaded area in millimetres for each of the 6 muscle directions below for the right eye and then the left eye:

Lateral rectus – at 0º
Superior rectus- at 67º
Inferior oblique – at 141º
Medial rectus – at 180º
Superior Oblique – at 216º
Inferior rectus – at 293º
(see below)

Distance to be measured shown by arrow for lateral rectus below:

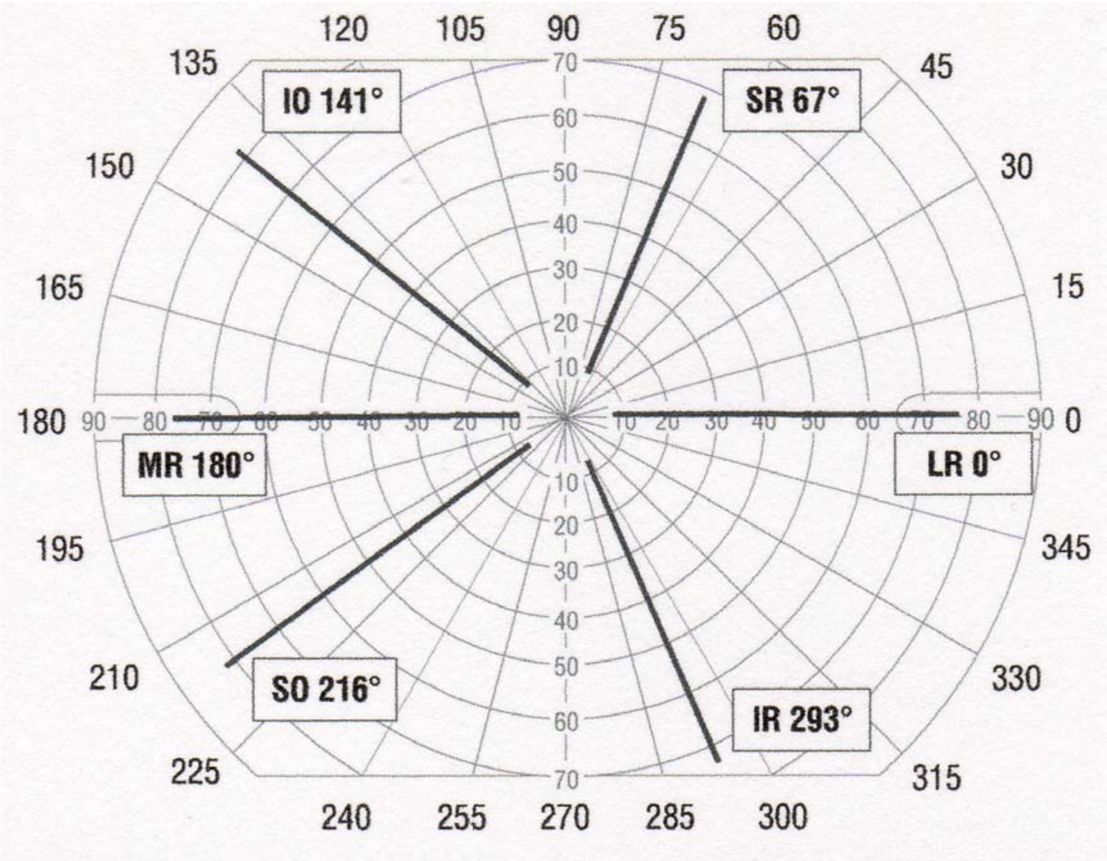

- Multiply each distance in millimetres by 0.83 and enter the value in the corresponding box in the source document and case report form.

UNIOCULAR FIELDS of FIXATION	right eye	left eye
Lateral rectus (0º)	o	o
Superior rectus (67º)	o	o
Inferior oblique (141º)	o	o
Medial rectus (180º)	o	o
Superior oblique (216º)	o	o
Inferior rectus (293º)	o	o

+Haggerty H, Richardson,S, Mitchell,KW, Dickinson,AJ. A modified method for measuring uniocular fields of fixation: reliability in healthy subjects and in patients with Graves orbitopathy. Archives of ophthalmology 2005;123(3):356-62.

Calculating Disease Activity (Clinical Activity Score[+])

Each feature below that is present counts as one point. The scores for each eye are calculated separately. The maximum score is 10 at each follow-up visit and 7 at enrolment (because there will be no previous records to determine changing measurements of proptosis, visual acuity and eye movements). The score for the worst eye is used.

Pain

- Pain on eye movement in the last 4 weeks
- Painful, oppressive feeling on or behind globe in the last 4 weeks

Redness

- Conjunctival redness
- Eyelid redness

Swelling

- Chemosis
- Swollen caruncle
- Eyelid oedema
- Increasing proptosis of > 2mm

Impaired Function

- Decreasing visual acuity of > 1 snellen line
- Decreasing eye movement of $\geq 8^{o}$ [53]

[+] Mourits M P, Prummel,MF, Wiersinga,WM, |Koornneef,L,. Clinical activity score as a guide in the management of patients with Graves' ophthalmopathy. Clinical endocrinology 1997;47(1):9-14.

Calculating Disease Severity (Total Eye Score*)

- Using the 'Total Eye Score Calculation Sheet' circle the corresponding value for each of the subclasses. For example, if the patient has eyelid swelling, conjunctival injection and chemosis this would equate to moderate soft tissue involvement and 4 would be circled. The score for each eye is calculated separately.

- Only circle 'only signs' if exophthalmometer readings are less than 23. 'Only signs' is unlikely to be relevant at the enrolment visit due to the inclusion criteria.

- When you have circled the five values (soft tissue involvement value, proptosis value, extraocular movement value, corneal value and visual acuity value), add them up to give you the total eye score for the left eye and then the total eye score for the right eye. The score for the worst eye (highest score) is recorded in the CRF.

- Total eye score must be completed on enrolment and on exit/final visit.

*Prummel MF, Mourits MP, Berghout A, Krenning EP, van der Gaag R, Koornneef L, et al. Prednisone and cyclosporine in the treatment of severe Graves' ophthalmopathy*New England Journal of Medicine* 1989;321(20):1353-9.

TOTAL EYE SCORE CALCULATION SHEET Patient Study No:…………………..

N	No signs or symptoms	0
O	Only signs – upper lid retraction and lid lag (only counted if proptosis <23mm)	1
S	Count the no. of the following present: eyelid swelling, conj redness, chemosis, lateral rectus insertion redness, caruncle inflammation	
	Absent soft tissue involvement (0)	0
	Minimal soft tissue involvement (1)	2
	Moderate soft tissue involvement (2 or 3)	4
	Marked soft tissue involvement (4 or 5)	6
P	<23mm proptosis	0
	23-24mm proptosis	3
	25-27mm proptosis	6
	≥ 28mm proptosis	9
E	Absent EOM involvement	0
	Limitation in extremes (inconstant diplopia)	4
	Evident restriction of movement (constant diplopia but globe not fixed)	8
	Fixation of globes	12
C	Absent corneal involvement	0
	Stippling/staining of cornea	5
S	Visual acuity > 6/9	0
	Visual acuity = 6/9 -6/18	6
	Visual acuity (6/18) – 6/60	12
	Visual acuity <6/60	18

Ring the relevant value in each class and add them up to give the Total Eye Score
Calculate score for right eye and left eye separately.
