# Supplementary material for: Protocol for the combined immunosuppression & radiotherapy in thyroid eye disease (CIRTED) trial: A multi-centre, double-masked, factorial randomised controlled trial
Source: Trials. 2008 Jan 31;9:6. doi: 10.1186/1745-6215-9-6 (PMC2275219; doi:10.1186/1745-6215-9-6)
Supplement: Additional file 4 — Case Report Form. Case report form used in the combined immunosuppression & radiotherapy in thyroid eye disease (CIRTED) trial. [file 1745-6215-9-6-S4.pdf]

PATIENT Initials: □□□

PATIENT Study No: □□□

CASE REPORT FORM

The CIRTED Study: Combined Immunosuppression and Radiotherapy in Thyroid Eye Disease

TRIAL LOG

1. **DATE ENROLLED: ____/____/____**

2. **DATE RANDOMISED: ____/____/____**

3. **DATE COMPLETED: ____/____/____**

IF WITHDRAWN EARLY
DATE OF WITHDRAWAL ____/____/____

COMPLETE STUDY WITHDRAWAL/COMPLETION FORM

HISTORY

Date TED diagnosed by clinician / clinical features first documented : (mm/yyyy):	…../………
Date Thyroid disorder diagnosed (mm/yyyy):	…../………
Thyroid status at thyroid diagnosis:	Hypothyroid ❑<br>Hyperthyroid ❑<br>Euthyroid ❑
Thyroid status at enrolment:	Hypothyroid ❑<br>Hyperthyroid ❑<br>Euthyroid ❑
Most recent Thyroid Function Tests prior to enrolment<br>Date:……/……./…….	fT4 ………… pmol/L<br>fT3 ………… pmol/L<br>TSH…………mU/L
Number of months biochemically euthyroid prior to enrolment<br>(if dysthyroid enter 0):	……………………
Previous steroid use of ≥20mg in last 6 months	YES / NO
Smoker at time of TED diagnosis	YES / NO

Visit 1 Enrolment visit

Date: ____/____/____

Week -2

PATIENT DEMOGRAPHICS

Patient Initials	☐☐☐
Date of Birth	☐☐ ☐☐ ☐☐☐☐<br>d m y
Sex	☐ male ☐ female
Ethnic Group	☐ Caucasian<br>☐ Black<br>☐ Oriental<br>☐ Asian<br>☐ Other (please specify)

Patient Study No:…………

INCLUSION CRITERIA

		No	Yes
1.	Mourits' Clinical Activity Score ≥ 4 (worst eye) <u>OR</u> ≥ 2 (worst eye) with a history of proptosis* or motility restriction† of less than 6 months duration	☐	☐
2.	Past or present history of abnormal Thyroid Gland Function <u>OR</u> a clinical diagnosis of TED made <u>and</u> confirmed by ≥ 2 muscle involvement on CT or MRI scan	☐	☐

EXCLUSION CRITERIA

All items must be checked 'no' for the patient to be eligible for entry into the study.

		No	Yes
1.	Age <20yrs old or > 75 yrs old	☐	☐
2.	Dysthyroid optic neuropathy	☐	☐
3.	Mourits' Clinical Activity Score > 4 without proptosis * or motility restriction†	☐	☐
4.	Use of radioiodine within the last 3 months	☐	☐
5.	Previous orbital irradiation	☐	☐
6.	Pre-existing glaucoma with a visual field defect & optic disc changes	☐	☐
7.	Pre-existing diabetes mellitus (not steroid induced)	☐	☐
8.	Previous adverse event associated with, or contraindication to, either Prednisolone or azathioprine	☐	☐
9.	Patient is within 6 months of pregnancy or planning pregnancy	☐	☐
10.	Lactation	☐	☐
11.	Haemoglobin Concentration > 1g/dl below local lab reference range, Total White Cell Count below local lab reference range or Platelet Count <130 X 10^9 /L	☐	☐
12.	Lymphocyte Count < 0.8 x 10^9 / L	☐	☐
13.	AbnormalThiopurine Methyltransferase (TPMT) activity	☐	☐
14.	Abnormal renal function (estimated Glomerular Filtration Rate eGFR < 60ml/min/1.73m^2)	☐	☐
15.	Abnormal liver function, specifically: bilirubin, alanine aminotransferase or alkaline phosphatase concentrations > 1.5 X above the local lab reference ranges	☐	☐
16.	Malignant or pre-malignant (dysplastic) condition within the past 5 years	☐	☐
17.	Previous tuberculosis	☐	☐
18.	Shingles within the past 3 months	☐	☐
19.	HIV / AIDS	☐	☐
20.	Concurrent use of other immunosuppressants/cytotoxic drugs/allopurinol	☐	☐
21.	Live vaccines within 3 months	☐	☐

* Definition of proptosis = EITHER subjective unilateral proptosis confirmed by asymmetry in exophthalmometry of ≥ 2mm OR subjective bilateral proptosis.

† Definition of motility restriction = intermittent, inconstant or constant diplopia grade

Enrolment visit clinical findings:

WEIGHT (kg)	BLOOD PRESSURE (mmHg)
□□□	□□□ / □□□

SYMPTOMS	
Pain or oppressive feeling behind globe within last 4 weeks	YES / NO
Pain on eye movement during the last 4 weeks	YES / NO
Gorman diplopia score	None - Intermittent - Inconstant - Constant

	RIGHT	LEFT
Best Corrected Snellen Visual Acuity:		
Eyelid swelling	YES / NO	YES / NO
Eyelid Erythema	YES / NO	YES / NO
Conjunctival redness	YES / NO	YES / NO
Lateral rectus injection	YES / NO	YES / NO
Chemosis	YES / NO	YES / NO

**Caruncle swelling**	**YES / NO**	**YES / NO**
**Corneal Pathology**	**Absent**<br>**Stippling**<br>**Ulcer**<br>**Perforation**	**Absent**<br>**Stippling**<br>**Ulcer**<br>**Perforation**
**IOP in primary position (mmHg)**		
**IOP in Upgaze (mmHg)**		
**Palpebral aperture**	**…………mm**	**…………mm**
**Fixed Globe**	**YES / NO**	**YES / NO**

EXOPHTHALMOMETRY		
**…………mm**	**…………mm**	**…………mm**

UNIOCULAR FIELDS of FIXATION		
Lateral rectus (0º)	º	º
Superior rectus (67º)	º	º
Inferior oblique (141º)	º	º
Medial rectus (180º)	º	º
Superior oblique (216º)	º	º
Inferior rectus (293º)	º	º

CLINICAL ACTIVITY SCORE AT ENROLMENT	
TOTAL EYE SCORE AT ENROLMENT<br><br>(see calculation sheet)	

I have reviewed all the enrolment data and found them to be complete and accurate.

Date and Investigator's Signature:____________________________________

Visit 2

Randomisation Visit

Date: ____/____/____

Week 0

Visit 2 clinical findings:

WEIGHT (kg)	BLOOD PRESSURE (mmHg)
□□□	□□□ / □□□

SYMPTOMS	
Pain or oppressive feeling behind globe within last 4 weeks	YES / NO
Pain on eye movement during the last 4 weeks	YES / NO
Gorman diplopia score	None - Intermittent - Inconstant - Constant

	RIGHT	LEFT
Best Corrected Snellen Visual Acuity:		
Eyelid swelling	YES / NO	YES / NO
Eyelid Erythema	YES / NO	YES / NO
Conjunctival redness	YES / NO	YES / NO
Lateral rectus injection	YES / NO	YES / NO
Chemosis	YES / NO	YES / NO

**Caruncle swelling**	**YES / NO**	**YES / NO**
**Corneal Pathology**	**Absent**<br>**Stippling**<br>**Ulcer**<br>**Perforation**	**Absent**<br>**Stippling**<br>**Ulcer**<br>**Perforation**
**IOP in primary position (mmHg)**		
**IOP in Upgaze (mmHg)**		
**Palpebral aperture**	**…………mm**	**…………mm**
**Fixed Globe**	**YES / NO**	**YES / NO**

EXOPHTHALMOMETRY		
**…………mm**	**…………mm**	**…………mm**

UNIOCULAR FIELDS of FIXATION		
Lateral rectus (0º)	º	º
Superior rectus (67º)	º	º
Inferior oblique (141º)	º	º
Medial rectus (180º)	º	º
Superior oblique (216º)	º	º
Inferior rectus (293º)	º	º

I have reviewed all the data and found them to be complete and accurate.

Date and Investigator's Signature:______________________________________

Visit 3

Date: ____/____/____

Week ……….

Visit type

Scheduled visit ☐

Extra visit ☐

6 weeks post radiotherapy visit ☐

Final visit ☐

Visit 3 clinical findings:

WEIGHT (kg)	BLOOD PRESSURE (mmHg)
□□□	□□□ / □□□

SYMPTOMS	
Pain or oppressive feeling behind globe within last 4 weeks	YES / NO
Pain on eye movement during the last 4 weeks	YES / NO
Gorman diplopia score	None - Intermittent - Inconstant - Constant

	RIGHT	LEFT
Best Corrected Snellen Visual Acuity:		
Eyelid swelling	YES / NO	YES / NO
Eyelid Erythema	YES / NO	YES / NO
Conjunctival redness	YES / NO	YES / NO
Lateral rectus injection	YES / NO	YES / NO
Chemosis	YES / NO	YES / NO

**Caruncle swelling**	**YES / NO**	**YES / NO**
**Corneal Pathology**	**Absent**<br>**Stippling**<br>**Ulcer**<br>**Perforation**	**Absent**<br>**Stippling**<br>**Ulcer**<br>**Perforation**
**IOP in primary position (mmHg)**		
**IOP in Upgaze (mmHg)**		
**Palpebral aperture**	**…………mm**	**…………mm**
**Fixed Globe**	**YES / NO**	**YES / NO**

EXOPHTHALMOMETRY		
**…………mm**	**…………mm**	**…………mm**

UNIOCULAR FIELDS of FIXATION		
Lateral rectus (0º)	º	º
Superior rectus (67º)	º	º
Inferior oblique (141º)	º	º
Medial rectus (180º)	º	º
Superior oblique (216º)	º	º
Inferior rectus (293º)	º	º

I have reviewed all the data and found them to be complete and accurate.

Date and Investigator's Signature:______________________________________

Visit 4

Date: ____/____/____

Week ……….

Visit type

Scheduled visit ☐

Extra visit ☐

6 weeks post radiotherapy visit ☐

Final visit ☐

Visit 4 clinical findings:

WEIGHT (kg)	BLOOD PRESSURE (mmHg)
□□□	□□□ / □□□

SYMPTOMS	
Pain or oppressive feeling behind globe within last 4 weeks	YES / NO
Pain on eye movement during the last 4 weeks	YES / NO
Gorman diplopia score	None - Intermittent - Inconstant - Constant

	RIGHT	LEFT
Best Corrected Snellen Visual Acuity:		
Eyelid swelling	YES / NO	YES / NO
Eyelid Erythema	YES / NO	YES / NO
Conjunctival redness	YES / NO	YES / NO
Lateral rectus injection	YES / NO	YES / NO
Chemosis	YES / NO	YES / NO

**Caruncle swelling**	**YES / NO**	**YES / NO**
**Corneal Pathology**	**Absent<br>Stippling<br>Ulcer<br>Perforation**	**Absent<br>Stippling<br>Ulcer<br>Perforation**
**IOP in primary position (mmHg)**		
**IOP in Upgaze (mmHg)**		
**Palpebral aperture**	**…………mm**	**…………mm**
**Fixed Globe**	**YES / NO**	**YES / NO**

EXOPHTHALMOMETRY		
**…………mm**	**…………mm**	**…………mm**

UNIOCULAR FIELDS of FIXATION		
Lateral rectus (0º)	º	º
Superior rectus (67º)	º	º
Inferior oblique (141º)	º	º
Medial rectus (180º)	º	º
Superior oblique (216º)	º	º
Inferior rectus (293º)	º	º

6 WEEK POST RADIOTHERAPY CLINICAL ACTIVITY SCORE	

I have reviewed all the data and found them to be complete and accurate.

Date and Investigator's Signature:_______________________________________

Visit 5

Date: ____/____/____

Week ……….

Visit type

Scheduled visit ☐

Extra visit ☐

6 weeks post radiotherapy visit ☐

Final visit ☐

Visual 5 clinical findings:

WEIGHT (kg)	BLOOD PRESSURE (mmHg)
□□□	□□□ / □□□

SYMPTOMS	
Pain or oppressive feeling behind globe within last 4 weeks	YES / NO
Pain on eye movement during the last 4 weeks	YES / NO
Gorman diplopia score	None - Intermittent - Inconstant - Constant

	RIGHT	LEFT
Best Corrected Snellen Visual Acuity:		
Eyelid swelling	YES / NO	YES / NO
Eyelid Erythema	YES / NO	YES / NO
Conjunctival redness	YES / NO	YES / NO
Lateral rectus injection	YES / NO	YES / NO
Chemosis	YES / NO	YES / NO

**Caruncle swelling**	**YES / NO**	**YES / NO**
**Corneal Pathology**	**Absent**<br>**Stippling**<br>**Ulcer**<br>**Perforation**	**Absent**<br>**Stippling**<br>**Ulcer**<br>**Perforation**
**IOP in primary position (mmHg)**		
**IOP in Upgaze (mmHg)**		
**Palpebral aperture**	**…………mm**	**…………mm**
**Fixed Globe**	**YES / NO**	**YES / NO**

EXOPHTHALMOMETRY		
**…………mm**	**…………mm**	**…………mm**

UNIOCULAR FIELDS of FIXATION		
Lateral rectus (0º)	º	º
Superior rectus (67º)	º	º
Inferior oblique (141º)	º	º
Medial rectus (180º)	º	º
Superior oblique (216º)	º	º
Inferior rectus (293º)	º	º

I have reviewed all the data and found them to be complete and accurate.

Date and Investigator's Signature:____________________________________

Visit 6

Date: ____/____/____

Week ……….

Visit type

Scheduled visit	☐
Extra visit	☐
6 weeks post radiotherapy visit	☐
Final visit	☐

Visit 6 clinical findings:

WEIGHT (kg)	BLOOD PRESSURE (mmHg)
☐☐☐	☐☐☐ / ☐☐☐

SYMPTOMS	
Pain or oppressive feeling behind globe within last 4 weeks	YES / NO
Pain on eye movement during the last 4 weeks	YES / NO
Gorman diplopia score	None - Intermittent - Inconstant - Constant

	RIGHT	LEFT
Best Corrected Snellen Visual Acuity:		
Eyelid swelling	YES / NO	YES / NO
Eyelid Erythema	YES / NO	YES / NO
Conjunctival redness	YES / NO	YES / NO
Lateral rectus injection	YES / NO	YES / NO
Chemosis	YES / NO	YES / NO

**Caruncle swelling**	**YES / NO**	**YES / NO**
**Corneal Pathology**	**Absent**<br>**Stippling**<br>**Ulcer**<br>**Perforation**	**Absent**<br>**Stippling**<br>**Ulcer**<br>**Perforation**
**IOP in primary position (mmHg)**		
**IOP in Upgaze (mmHg)**		
**Palpebral aperture**	**…………mm**	**…………mm**
**Fixed Globe**	**YES / NO**	**YES / NO**

EXOPHTHALMOMETRY		
**…………mm**	**…………mm**	**…………mm**

UNIOCULAR FIELDS of FIXATION		
Lateral rectus (0º)	º	º
Superior rectus (67º)	º	º
Inferior oblique (141º)	º	º
Medial rectus (180º)	º	º
Superior oblique (216º)	º	º
Inferior rectus (293º)	º	º

I have reviewed all the data and found them to be complete and accurate.

Date and Investigator's Signature:____________________________________

Visit 7

Date: ____/____/____

Week   ……….

Visit type

Scheduled visit ☐

Extra visit ☐

6 weeks post radiotherapy visit ☐

Final visit ☐

Visit 7 clinical findings:

WEIGHT (kg)	BLOOD PRESSURE (mmHg)
□□□	□□□ / □□□

SYMPTOMS	
Pain or oppressive feeling behind globe within last 4 weeks	YES / NO
Pain on eye movement during the last 4 weeks	YES / NO
Gorman diplopia score	None - Intermittent - Inconstant - Constant

	RIGHT	LEFT
Best Corrected Snellen Visual Acuity:		
Eyelid swelling	YES / NO	YES / NO
Eyelid Erythema	YES / NO	YES / NO
Conjunctival redness	YES / NO	YES / NO
Lateral rectus injection	YES / NO	YES / NO
Chemosis	YES / NO	YES / NO

**Caruncle swelling**	**YES / NO**	**YES / NO**
**Corneal Pathology**	**Absent**<br>**Stippling**<br>**Ulcer**<br>**Perforation**	**Absent**<br>**Stippling**<br>**Ulcer**<br>**Perforation**
**IOP in primary position (mmHg)**		
**IOP in Upgaze (mmHg)**		
**Palpebral aperture**	**…………mm**	**…………mm**
**Fixed Globe**	**YES / NO**	**YES / NO**

EXOPHTHALMOMETRY		
**…………mm**	**…………mm**	**…………mm**

UNIOCULAR FIELDS of FIXATION		
Lateral rectus (0º)	º	º
Superior rectus (67º)	º	º
Inferior oblique (141º)	º	º
Medial rectus (180º)	º	º
Superior oblique (216º)	º	º
Inferior rectus (293º)	º	º

SUBJECTIVE EYE SCORE AT FINAL VISIT		
Improved	Unchanged	Worsened

TOTAL EYE SCORE AT FINAL VISIT	

I have reviewed all the data and found them to be complete and accurate.

Date and Investigator's Signature:______________________________________

ADVERSE EVENT FORM

All adverse events and serious adverse events must be recorded below.

AE No	Diagnosis or signs/symptoms	SAE code	Date Started<br>D m y	Date Stopped<br>d m y	Outcome	Maximum intensity	Site of infection	Type of infection
			□□ □□ □□□□	□□ □□ □□□□				
			□□ □□ □□□□	□□ □□ □□□□				
			□□ □□ □□□□	□□ □□ □□□□				
			□□ □□ □□□□	□□ □□ □□□□				
			□□ □□ □□□□	□□ □□ □□□□				
			□□ □□ □□□□	□□ □□ □□□□				

SAE CODE:	OUTCOME	MAXIMUM INTENSTIY	SITE OF INFECTION: (more than one possible)	TYPE OF INFECTION:
0 = not serious<br>1 = patient dies<br>2 = life threatening<br>3 = involved or prolonged in-patient hospitalisation<br>4 = Involved persistent or significant disability or incapacity<br>5 = congenital abnormality<br>6 = intervention required to prevent one of the above<br><br>NOTE: If 1 – 5 is recorded, please complete SERIOUS ADVERSE EVENT REPORT form	1 = recovered<br>2 = recovered with residual effect(s)<br>3 = ongoing<br>4 = death<br>5 = ongoing at time of death<br>6 = unknown	1 = mild<br>2 = moderate<br>3 = severe	0 = no infection<br>1 = systemic<br>2 = cardiovascular<br>3 = respiratory<br>4 = gastrointestinal<br>5 = urogenital<br>6 = musculoskeletal<br>7 = nervous system<br>8 = skin<br>9 = ears, nose, throat<br>10 = eyes<br>11 = abdominal<br>12 = other	0 = no infection<br>1 = unknown<br>2 = bacterial<br>3 = viral<br>4 = fungal<br>5 = protozoal<br>6 = other

Patient Study No:…………

ADVERSE EVENT FORM

All adverse events and serious adverse events must be recorded below.

AE No	Diagnosis or signs/symptoms	SAE code	Date Started D m y	Date Stopped d m y	Outcome	Maximum intensity	Site of infection	Type of infection
			☐☐ ☐☐ ☐☐☐☐	☐☐ ☐☐ ☐☐☐☐				
			☐☐ ☐☐ ☐☐☐☐	☐☐ ☐☐ ☐☐☐☐				
			☐☐ ☐☐ ☐☐☐☐	☐☐ ☐☐ ☐☐☐☐				
			☐☐ ☐☐ ☐☐☐☐	☐☐ ☐☐ ☐☐☐☐				
			☐☐ ☐☐ ☐☐☐☐	☐☐ ☐☐ ☐☐☐☐				
			☐☐ ☐☐ ☐☐☐☐	☐☐ ☐☐ ☐☐☐☐				

SAE CODE:	OUTCOME	MAXIMUM INTENSTIY	SITE OF INFECTION: (more than one possible)	TYPE OF INFECTION:
0 = not serious<br>1 = patient dies<br>2 = life threatening<br>3 = involved or prolonged in-patient hospitalisation<br>4 = Involved persistent or significant disability or incapacity<br>5 = congenital abnormality<br>6 = intervention required to prevent one of the above<br><br>NOTE: If 1 – 5 is recorded, please complete SERIOUS ADVERSE EVENT REPORT form	1 = recovered<br>2 = recovered with residual effect(s)<br>3 = ongoing<br>4 = death<br>5 = ongoing at time of death<br>6 = unknown	1 = mild<br>2 = moderate<br>3 = severe	0 = no infection<br>1 = systemic<br>2 = cardiovascular<br>3 = respiratory<br>4 = gastrointestinal<br>5 = urogenital<br>6 = musculoskeletal<br>7 = nervous system<br>8 = skin<br>9 = ears, nose, throat<br>10 = eyes<br>11 = abdominal<br>12 = other	0 = no infection<br>1 = unknown<br>2 = bacterial<br>3 = viral<br>4 = fungal<br>5 = protozoal<br>6 = other

ADDITIONAL INFORMATION eg date of start & finish of radiotherapy / protocol deviation / concomitant procedures

DATE	TYPE OF INFORMATION eg protocol deviation	INFORMATION	OUTCOME

STUDY WITHDRAWAL FORM

Did the patient discontinue the trial prematurely? ☐ no ☐ yes*

If 'Yes', enter the reason for the study discontinuation.

Date of premature Study Discontinuation: ☐☐ ☐☐ ☐☐☐☐
d m y

Primary Reason for Discontinuation:
(tick one box only)

- ☐ Patient withdraws consent
  If known, state reason: ____________________
- ☐ Patient is non-compliant
- ☐ Patient is pregnant
- ☐ Patient is lost to follow up
- ☐ Lack of efficacy of treatment
- ☐ Investigator feels that it is in the patient's best interest due to an adverse event

Related AE No.: __________

Other (specify): ____________________ ____________________

Investigator's Signature: ____________________

STUDY COMPLETION FORM

Did the patient complete the trial? ☐ no ☐ yes

If 'No', complete study withdrawal form

Date of Study Completion:	☐☐ ☐☐ ☐☐☐☐<br>d m y

Investigator's Signature: ______________________________
